# Single base substitution mutational signatures in pediatric acute myeloid leukemia based on whole genome sequencing

**DOI:** 10.1038/s41375-021-01242-0

**Published:** 2021-04-16

**Authors:** Rebeqa Gunnarsson, Minjun Yang, Linda Olsson-Arvidsson, Andrea Biloglav, Mikael Behrendtz, Anders Castor, Kajsa Paulsson, Bertil Johansson

**Affiliations:** 1 Division of Clinical Genetics, Department of Laboratory Medicine, Lund University, Lund, Sweden; 2 Department of Clinical Genetics and Pathology, Division of Laboratory Medicine, Lund, Sweden; 3 Department of Pediatrics, Linköping University Hospital, Linköping, Sweden; 4 Department of Pediatrics, Skåne University Hospital, Lund, Sweden

**Keywords:** Cancer genomics, Acute myeloid leukaemia

## To the Editor:

Whole genome sequencing (WGS) of neoplasms not only offers a comprehensive genome-wide detection of gene fusions/other structural variants (SVs), gene mutations, and copy number abnormalities (CNAs) but also provides information on acquired mutational profiles, including localized hypermutations (kataegis) and single base substitution (SBS) mutational signatures. The latter are recurring trinucleotide patterns of somatic single nucleotide variants (SNVs) and their flanking nucleotides that are, in some instances, associated with the etiology or pathogenesis of neoplastic disorders, for example C>T transitions in UV-associated melanoma, C>A transversions in smoking-induced lung cancer, and C>T transitions and C>G transversions caused by overactive APOBEC enzymes [1, 2]. In addition, WGS allows detection of variants in non-coding regulatory elements (REs), such as enhancers and promoters, resulting in de-regulated gene transcription [3]. However, the few WGS studies of pediatric acute myeloid leukemia (AML) reported to date have not ascertained SBS mutational signatures or variants in REs [4–6]. We performed WGS on 20 pediatric AML cases (Supplementary Information and Supplementary Fig. S1), focusing on SBS profiles, variants in REs, and novel gene fusions and mutations.

Apart from confirming all gene fusions detected in clinical routine, the SV analysis revealed three novel in-frame fusions: *PLEKHA5-ADAMTS20*, *RAB11FIP2-NEURL4*, and *TCF3-HOXB9* (Supplementary Information, Supplementary Tables S1 and S2, and Supplementary Figs. S2 and S3). The *TCF3-HOXB9* fusion recombines two transcription factor genes that are important for differentiation, such as lymphopoiesis, and proliferation and that are expressed in bone marrow (BM) and lymph nodes (LNs) (Supplementary Table S2). In addition, *TCF3* is a partner in several other fusion genes in, *e.g*., acute lymphoblastic leukemia (ALL). The *PLEKHA5-ADAMTS20* fusion involves two genes that are often fused to other genes in, mainly, epithelial malignancies but that are also normally expressed in BM and LNs. (Supplementary Table S2). *RAB11FIP2-NEURL4* rearranges *RAB11FIP2*, expressed in BM and LNs, with the mitotic gene *NEURL4*, which is fused to, for example, *MSI2* in malignant melanoma (supplementary Table 2). An *XPO1-TNRC18* fusion, previously reported in a single case of ALL [7], was also identified. In total, 13 (65%) of the 20 AMLs harbored WGS-identified fusion genes (Supplementary Table S1). Although we cannot exclude the possibility that the novel fusions were merely byproducts of the genetic instability present in many tumor types—such “passenger fusions” are commonly identified by various types of massively parallel sequencing [8]—we consider this unlikely. First, all gene partners in the novel fusions, except *RAB11FIP2*, have previously been reported to be rearranged with other genes in human malignancies (Supplementary Table S2; https://mitelmandatabase.isb-cgc.org/); this increases the likelihood that they are pathogenetically important. Second, the fact that the fusion genes were identified by WGS clearly shows that they existed on the DNA level and, hence, were not transcription-induced [8, 9].

**Fig. 1:**
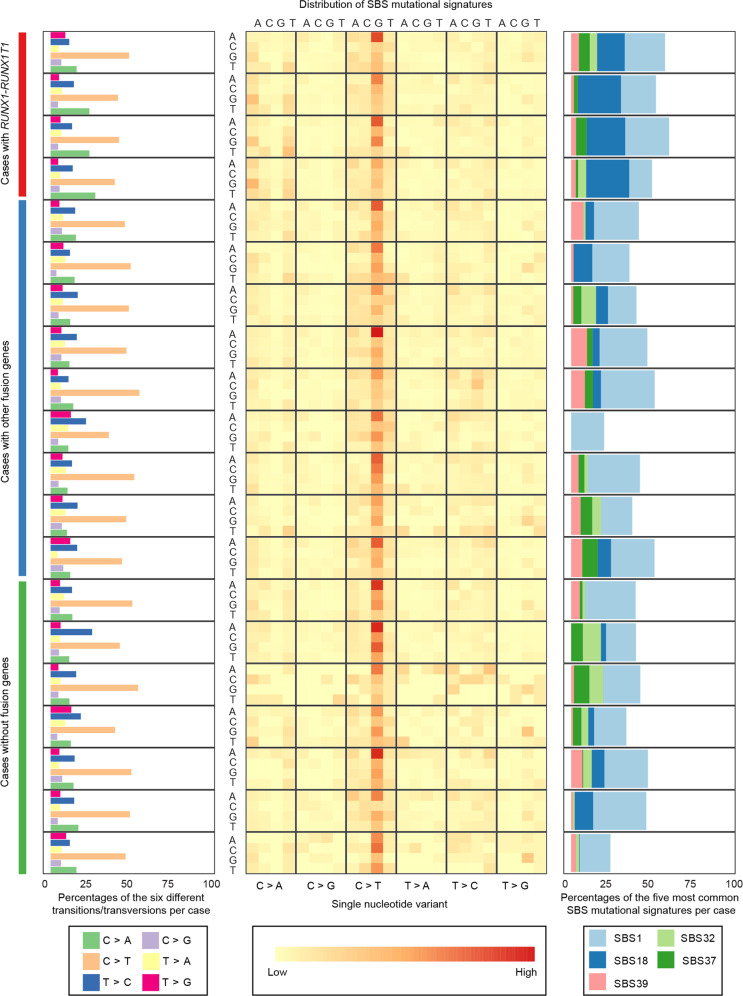
Overview of the transition and transversion types and the single base substitution (SBS) mutational signatures in the pediatric acute myeloid leukemias cases. Left panel: the distribution of the six different transition and transversion types per case. Middle panel: heat map showing the distribution of the SBS signatures in each case. Right panel: The frequencies of the five overall most common signatures (SBS1, SBS18, SBS32, SBS37, and SBS39) per case.

A total of 34 CNAs (24 losses and 10 gains; median 1.5 CNAs/case) and two uniparental isodisomies were detected; none of the cases displayed chromothripsis (Supplementary Information and Supplementary Table S3). The frequencies of imbalances did not differ between cases with or without (w/wo) fusion genes (*P* = 0.3). Of the 10 920 somatic variants identified (Supplementary Table S1), 10,492 (96%) were SNVs and 428 (4%) small insertions/deletions (indels). There were between 73 and 1 198 SNVs/indels per case (median 502/case), corresponding to 0.024-0.386 SNVs/indels per Mb. This low frequency of SNVs/indels agrees well with a previous study showing that pediatric AMLs harbor fewer mutations than other childhood cancers [10]. Furthermore, the rainfall plot analyses revealed kataegic regions in only two cases (Supplementary Fig. S4). The most common type of SNV was a C>T transition (48%), followed by C>A transversions (17%), T>C transitions (15%), T>G (7%), T>A (7%), and C>G (6%) transversions (Fig. 1). All transitions and transversions, except C>A, increased significantly with age (Supplementary Fig. S5), as expected considering that many acquired SNVs are known to occur in a clockwise manner [11]. Neither the total mutational burden nor the different types of substitution differed significantly between cases w/wo fusion genes (data not shown).

The SBS mutational signature analysis revealed that signature 1 (SBS1) was the most common one, detected in all 20 AMLs with relative contributions of 15–34% per case (median 25%; Fig. 1). It is associated with a predominance of C>T transitions resulting from endogenous, spontaneous deamination of 5-methylcytosines and increasing by age [1]. The second most frequent signature was SBS18, found in 15 of the AMLs (median relative contribution of 8.2%), which is characterized by C>A transversions and possibly associated with damage caused by reactive oxygen species (ROS) (https://cancer.sanger.ac.uk/cosmic/signatures/SBS/SBS18.tt). The SBS39, SBS37, and SBS32 signatures were detected in 18, 15, and 13 of the cases, with median values of relative contributions of 4.0%, 3.9%, and 3.2%, respectively. The etiologies of SBS37 and SBS39 are unknown, whereas SBS32 has been associated with prior treatment with azathioprine (https://cancer.sanger.ac.uk/cosmic/signatures/SBS). In the hierarchical cluster analysis of the above-mentioned signatures, the *RUNX1-RUNXT1*-positive cases clustered together in a separate branch, most likely because of the high proportions of SBS18 signatures/C>A transversions in these cases (Supplementary Fig. S6). Interestingly, frequent C>A transversions in *RUNX1-RUNXT1*-positive cases have previously been reported [4] and, although SBS mutational profiling was not performed in that study, it supports the association between C>A transversions, SBS18, and t(8;21) AML identified herein. In order to validate the association between *RUNX1*-*RUNX1T1*, C>A transversions, and SBS18, we utilized the TCGA dataset [12]; however, it should be stressed that this is based on whole exome sequencing data and hence is much less informative than WGS data with regard to number of SNVs. First, we compared the frequencies of the different SBS types in *RUNX1*-*RUNX1T1*-positive and -negative cases in our cohort and in the TCGA dataset. As seen in Supplementary Table S4, a relatively high frequency of C>A transversions was observed in the *RUNX1-RUNXT1*-positive cases also in the TCGA cohort. Furthermore, the SBS18 signature was observed in the *RUNX1-RUNX1T1*-positive group in the TCGA dataset; in contrast, no SBS18 signature was identified in the *RUNX1-RUNX1T1*-negative subgroups (Supplementary Table S5). Although based on whole exome sequencing data, the TCGA cohort thus provides some support for the association between C>A transversions, SBS18, and t(8;21) in AML.

Comparing the SNVs/indels found at diagnosis and relapse of cases 1 and 2 revealed that 51% (case 1) and 33% (case 2) of the SNVs/indels at relapse were identical to those detected at diagnosis; the other SNVs/indels were either novel or lost at relapse (Supplementary Fig. S7). Interestingly, the SBS mutational signatures were similar at diagnosis and relapse (data no shown), showing that the induction and consolidation therapies did not result in different signatures at relapse.

**Fig. 2:**
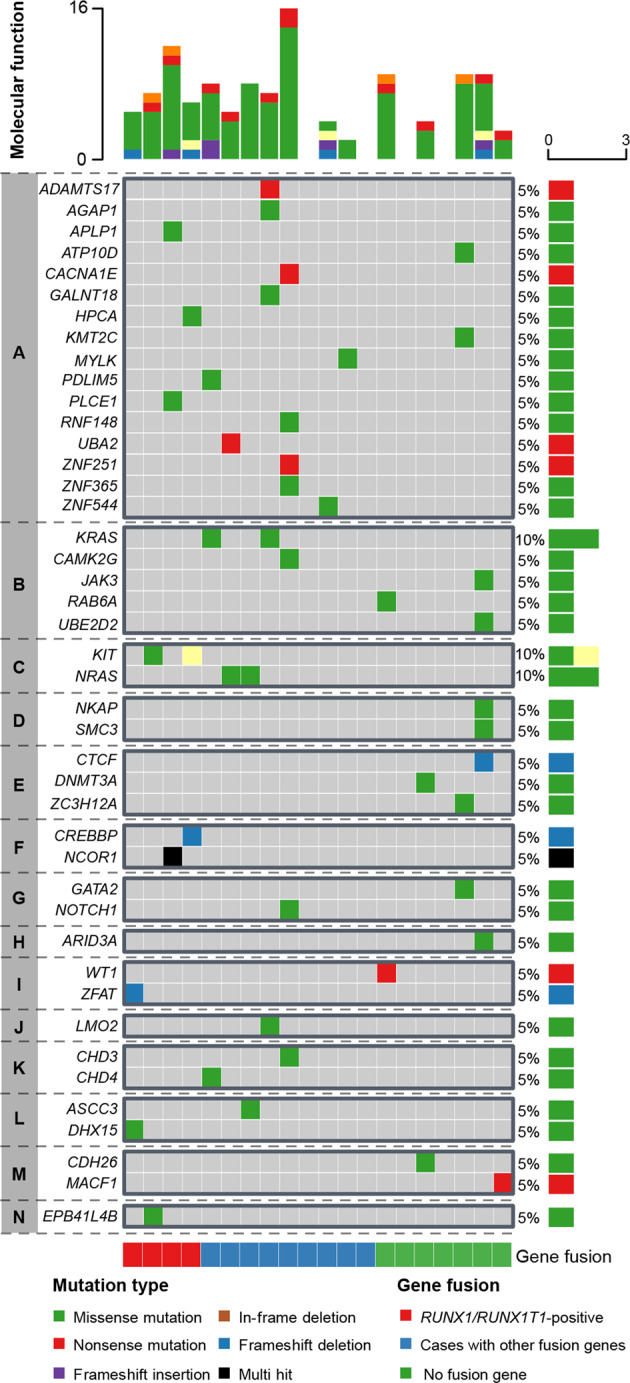
Overview of gene ontology (GO)-annotated molecular functions of the 43 genes with pathogenic variants. **A**–**N** indicate different GO functions: **A** metal ion binding; **B** nucleotide binding; **C** metal ion and nucleotide binding; **D** chromatin binding; **E** metal ion and chromatin binding; **F** chromatin and polymerase II activating transcription factor binding; **G** metal ion, chromatin/DNA binding, and transcription activator; **H** chromatin/DNA binding and transcription activator; **I** metal ion, DNA binding, and transcription activator; **J** metal ion, DNA and RNA polymerase II activating transcription factor binding, and transcription activator; **K** metal ion binding and helicase activity; **L** helicase activity; **M** metal ion binding and cytoskeletal protein binding; **N** cytoskeletal protein binding.

Of the 10,920 SNVs/indels, 123 (1.1%) occurred within coding genes (Supplementary Table S6) and 110 of these could be confirmed by deep sequencing, whole exome sequencing, or Sanger sequencing. Eighty-nine of the verified SNVs/indels in 84 different genes were considered pathogenic, either by default by being truncating or by being classified as such by SIFT and/or PolyPhen. Gene ontology data on molecular functions were available for 43 of the 84 genes: the most frequent functions were transcriptional regulation (67%) and metal ion binding (67%) (Fig. 2 and Supplementary Fig. S8). Apart from genes previously reported to be mutated in pediatric AML, *e.g., DNMT3A*, *GATA2*, *JAK3*, *NCOR1*, and *NOTCH1* [4, 13], we identified pathogenic SNVs/indels in 33 genes (Supplementary Table S6) previously not implicated in AML, such as *RASL11A* (RAS signaling), *ATPB5* (mitochondrial metabolism), and *ASCC3*, *MACF1*, *USF2*, *ZFAT*, and *ZNF251* (transcriptional regulation).

The analysis of the 10,920 somatic SNVs/indels revealed that 133 (1.2%) of them occurred in REs, mainly enhancers (41%) and promoters (26%) (Supplementary Information and Supplementary Table S7). The potential targets of the RE variants comprised 656 targets—506 (77%) genes and 150 (23%) ncRNAs. Only six genes and one ncRNA were recurrently targeted (Supplementary Table S7), suggesting that the RE variants did not affect specific genes/pathways.

In conclusion, the present WGS analysis of pediatric AML identified both known and novel fusion genes (*PLEKHA5-ADAMTS20*, *RAB11FIP2-NEURL4*, and *TCF3-HOXB9*) and revealed relatively few CNAs and SNAs/indels as compared with other malignancies. Furthermore, regions with kataegis were rare and there were no signs of chromothripsis. Thus, childhood AML is characterized by a low degree of genomic complexity and mutational burden. Of the acquired variants detected, only a minority targeted REs (~1%) and coding genes (~1%). However, a large proportion (39%) of the coding genes had previously not been implicated in AML. Although fusion genes are considered strong driver mutations, requiring few additional hits for generating overt leukemia [14], this was not reflected by differences in CNAs, SNVs/indels, overall genomic complexity, functions of the mutated genes, or SBS mutational signatures between fusion-positive and -negative cases. However, *RUNX1-RUNXT1*-positive cases were for the first time associated with a higher prevalence of the ROS-associated SBS18 signature, likely due to the high frequency of C>A transversions in this AML subtype. Although the correlation between SBS18 and t(8;21) should be considered preliminary—additional studies are needed to confirm or refute this—the present finding indicates that DNA damage caused by ROS [14, 15] may be of particular importance in *RUNX1-RUNXT1*-positive AML.

## Supplementary information

Supplemental Material

Supplemental Information

## Data Availability

The dataset generated during the current study fall under the GDPR regulations for sharing of personal data and will therefore be made available in the EGA-SE depository upon its completion. Until then, the data are available from the corresponding author upon request through the following DOI: https://figshare.com/s/5a1ca3f39611c39bfaae (WGS dataset). Supplementary information is available at Leukemia’s website.
