# Supplementary material for: Single base substitution mutational signatures in pediatric acute myeloid leukemia based on whole genome sequencing
Source: Leukemia. 2021 Apr 16;35(5):1485–9. doi: 10.1038/s41375-021-01242-0 (PMC8102186; doi:10.1038/s41375-021-01242-0)
Supplement: Supplementary file 1 — Supplemental Material [file 41375_2021_1242_MOESM1_ESM.docx]

**Supplementary Information**

**Patient samples and clinical data**

Twenty diagnostic, 20 remission, and two relapse samples from 20 children/adolescents with acute myeloid leukemia (AML), treated at the Departments of Pediatrics at Lund and Linköping University Hospitals between 1994 and 2016, were analyzed by whole genome sequencing (WGS). The median age of the patients was 8 years (range 0-17 years) and the female/male ratio was 1:1. Apart from chromosome banding analysis at the time of diagnosis, fluorescence in situ hybridization or molecular genetic investigations for *KMT2A* rearrangements and the *CBFB*-*MYH11*, *PML*-*RARA*, and *RUNX1*-*RUNX1T1* gene fusions had been performed in all cases except in two *RUNX1*-*RUNX1T1*-positive AMLs. Sixteen of the cases had also been analyzed by single nucleotide polymorphism array and by deep sequencing of 100 leukemia-associated genes, as previously reported [1]. The basic clinical and genetic features of the cases are summarized in Supplementary Table 1.

The investigation was approved by the Research Ethics Committee of Lund University and informed consent for the analyses was obtained according to the Declaration of Helsinki.

**Whole genome sequencing**

DNA was extracted from diagnostic bone marrow (BM; *n* = 17)/peripheral blood (PB; *n* = 3) samples, remission BM (*n* = 15)/PB (*n* = 5), and from two BM relapses. Construction of libraries, using the TruSeq Nano DNA sample preparation kit (Illumina, San Diego, CA, USA) on 100 ng DNA, and massively parallel sequencing were performed by BGI Tech Solutions (Hong Kong). The WGS (Illumina HiSeqX) reached an average sequencing depth of 30-42x/sample (median 33x), with 2x 150 bp read length. Three of the cases (#3, 7, and 9; Supplementary Table 1) had also been analyzed in-house by whole exome sequencing (WES) using the Nextera Rapid Capture Expanded Exome Kit (Illumina), with a coverage of ~150x. The paired-end reads were aligned to the human reference genome GRCh37 (hg19) by the Burrows-Wheeler Aligner tool (version 0.7.17) [2]. Duplicate reads marking and local realignment were performed by GATK (version 4.0.11.0) [3].

**Sequence analyses: SVs, CNAs, SNVs/indels, and mutational profiles**

Somatic structural variants (SVs) were identified by Manta Structural Variant Caller [4], DELLY [5], novoBreak [6], and SvABA [7], with default settings, and then merged using MAVIS [8] (Supplementary Tables 1 and 2).

Patchwork [9] was applied to ascertain somatic copy number abnormalities (CNAs), *i.e.*, deletions, duplications, amplifications, chromothripsis, and whole chromosome gains and losses, as well as uniparental isodisomies (Supplementary Table 3).

MuTect (version 1.1.7) [10], Mutect2 (version 4.0.11.0) [10], and MuSE (version v1.0rc) [11] were used to identify somatic single nucleotide variants (SNVs) and small insertions/deletions (indels). The SNVs/indels called by Mutect2 were further filtered with the GATK module FilterMutectCalls [3]. The variant vcf files were converted into maf files by the vcf2maf package (<https://github.com/mskcc/vcf2maf>) and annotated by the Ensembl Variant Effect Predictor (<http://www.ensembl.org/info/docs/tools/vep/index.html>) [12]. All acquired SNVs/indels in coding genes are listed in Supplementary Table 6.

Regions with kataegis [13] were identified by Maftools [14], using default settings, and Maftools was also used for generation of rainfall plots.

Fusion genes and WES-identified somatic variants in the TCGA AML cohort were downloaded from cBioPortal (https://www.cbioportal.org/study/summary?id=laml_tcga_pub) [15]. The MAF (data_mutations_extended.txt) files were converted to VCF files by the vcf2maf package (<https://github.com/mskcc/vcf2maf>), after which the SNVs of all cases belonging either to the *RUNX1-RUNX1T1*-positive group or the *RUNX1-RUNX1T1*-negative group were merged using bcftools (http://samtools.github.io/bcftools/). The cases in each of the two groups had to be merged because of the relative rarity of WES-identified SNVs in the TCGA dataset compared with the much large number of SNVs detected by WGS.

The R package MutationalPatterns [16] was used to decompose mutational profiles into pre-defined single base substitution (SBS) mutational signatures based on the sanger mutational signatures (v3.1 - June 2020) [17] and to ascertain the relative contributions of the different SBS types in each case in the present WGS study and in the different gene fusion groups (*RUNX1*-*RUNX1T1*, *CBFB*-*MYH11*, *PML*-*RARA*, other fusions, and no fusions) and in the TCGA dataset. To analyze the frequencies of the SBS types, the number of SBSs were calculated in each case of the present study and in the merged SNVs data in the TCGA cohort. Hierarchical clustering was performed based on the relative contributions of the five most common mutational signature types across all the cases in the present WGS study, as determined by MutationalPatterns.

Variants in regulatory elements (REs) were annotated by dbSUPER [18], EnhancerAtlas [19], JEME [20], and by the Promoter Capture Hi-C (PCHi-C) dataset [21]. Non-coding variants located within REs, reported in hematologic cells (Supplementary Table 8) in at least two of the above-mentioned databases, were kept for further analysis. AML H3K27ac histone marks chromatin immunoprecipitation sequencing (ChIP-seq) data (https://www.ncbi.nlm.nih.gov/bioproject/PRJNA381888) were used to investigate whether the REs variants occurred in AML H3K27ac ChIP-seq peak regions, called by MACS2 with default settings. Only REs variants located within conserved functionally active regions (peak observed in ≥10 ChIP-seq samples) were selected. The putative functions of the remaining REs variants were evaluated according to chromatin states in ChromHMM [22].

**Verification of WGS data**

Four novel fusion genes (*NCAM2-RUNX1*, *PLEKHA5-ADAMTS20*, *RAB11FIP2-NEURL4*, and *TCF3-HOXB9*) were detected by WGS. Of these, *PLEKHA5-ADAMTS20*, *RAB11FIP2-NEURL4*, and *TCF3-HOXB9* could be confirmed by RT-PCR and Sanger sequencing (Supplementary Table 1). Forward and reverse primers (Supplementary Table 9) were designed with Primer3 (http://bioinfo.ut.ee/primer3/) and purchased from Thermo Fisher Scientific (Waltham, MA, USA). Chromas Lite 2.6 (Technelysium, South Brisbane, Australia) was used for sequence analysis. Despite several attempts, the *NCAM2-RUNX1* fusion could not be confirmed by RT-PCR. Thus, it was either a false positive fusion or there were too many structural variations in the breakpoint regions, precluding a correct design of primers.

A total of 123 SNVs and indels in exons were detected when using low (10-20 reads with ≥25% variant detection; *n* = 30) and high (>20 reads with ≥10% variant detection; *n* = 93) confidence filtering criteria. Of these, 97 were selected for verification by Sanger sequencing (the remaining variants were verified by already available deep sequencing [1] or WES data; Supplementary Table 6). Forward and reverse primers for Sanger sequencing (Supplementary Table 10) were designed with Primer3 (http://bioinfo.ut.ee/primer3/) and purchased from Thermo Fisher Scientific. Chromas Lite 2.6 was used for sequence analysis.

In cases 1 and 2 (Supplementary Table 1), relapse samples, received 10 and 9 months after diagnosis, respectively, were available and comparisons of the SNVs detected at diagnosis and at relapse were performed by vcftoolz and Venn diagrams were generated by vcftoolz compare (https://joss.theoj.org/papers/10.21105/joss.01144).

**Statistical tools and gene ontology analysis**

Regression analysis and two-tailed Wilcoxon signed-rank tests were performed by R, version 3.5.1 (https://www.r-project.org), using default settings. For gene ontology analysis of the genes with variants, GeneCards (https://geneanalytics.genecards.org/) was applied to extract information on molecular functions, biological processes, and protein classes [23].

**REFERENCES**

1. Olsson L, Zettermark S, Biloglav A, Castor A, Behrendtz M, Forestier E, et al. The genetic landscape of paediatric *de novo* acute myeloid leukaemia as defined by single nucleotide polymorphism array and exon sequencing of 100 candidate genes. Br J Haematol. 2016;174:292-301.

2. Li H, Durbin R. Fast and accurate short read alignment with Burrows-Wheeler transform. Bioinformatics. 2009;25:1754-60.

3. DePristo MA, Banks E, Poplin R, Garimella KV, Maguire JR, Hartl C, et al. A framework for variation discovery and genotyping using next-generation DNA sequencing data. Nat Genet. 2011;43:491-8.

4. Chen X, Schulz-Trieglaff O, Shaw R, Barnes B, Schlesinger F, Källberg M, et al. Manta: rapid detection of structural variants and indels for germline and cancer sequencing applications. Bioinformatics. 2016;32:1220-2.

5. Rausch T, Zichner T, Schlattl A, Stütz AM, Benes V, Korbel JO. DELLY: structural variant discovery by integrated paired-end and split-read analysis. Bioinformatics. 2012;28:i333-9.

6. Chong Z, Ruan J, Gao M, Zhou W, Chen T, Fan X, et al. novoBreak: local assembly for breakpoint detection in cancer genomes. Nat Methods. 2017;14:65-7.

7. Wala JA, Bandopadhayay P, Greenwald NF, O'Rourke R, Sharpe T, Stewart C, et al. SvABA: genome-wide detection of structural variants and indels by local assembly. Genome Res. 2018;28:581-91.

8. Reisle C, Mungall KL, Choo C, Paulino D, Bleile DW, Muhammadzadeh A, et al. MAVIS: merging, annotation, validation, and illustration of structural variants. Bioinformatics. 2019;35:515-7.

9. Mayrhofer M, DiLorenzo S, Isaksson A. Patchwork: allele-specific copy number analysis of whole-genome sequenced tumor tissue. Genome Biol. 2013;14:R24.

10. Cibulskis K, Lawrence MS, Carter SL, Sivachenko A, Jaffe D, Sougnez C, et al. Sensitive detection of somatic point mutations in impure and heterogeneous cancer samples. Nat Biotechnol. 2013;31:213-9.

11. Fan Y, Xi L, Hughes DST, Zhang J, Zhang J, Futreal PA, et al. MuSE: accounting for tumor heterogeneity using a sample-specific error model improves sensitivity and specificity in mutation calling from sequencing data. Genome Biol. 2016;17:178.

12. McLaren W, Gil L, Hunt SE, Riat HS, Ritchie GRS, Thormann A, et al. The Ensembl Variant Effect Predictor. Genome Biol. 2016;17:122.

13. Nik-Zainal S, Alexandrov LB, Wedge DC, Van Loo P, Greenman CD, Raine K, et al. Mutational processes molding the genomes of 21 breast cancers. Cell. 2012;149:979-93.

14. Mayakonda A, Lin D-C, Assenov Y, Plass C, Koeffler HP. Maftools: efficient and comprehensive analysis of somatic variants in cancer. Genome Res. 2018;28:1747-56.

15. Ley TJ, Miller C, Ding L, Raphael BJ, Mungall AJ, et al. Genomic and epigenomic landscapes of adult de novo acute myeloid leukemia. N Engl J Med. 2013;368:2059-74.

16. Blokzijl F, Janssen R, van Boxtel R, Cuppen E. MutationalPatterns: comprehensive genome-wide analysis of mutational processes. Genome Med. 2018;10:33.

17. Alexandrov LB, Kim J, Haradhvala NJ, Huang MN, Tian Ng AW, Wu Y, et al. The repertoire of mutational signatures in human cancer. Nature. 2020;578:94-101.

18. Khan A, Zhang X. dbSUPER: a database of super-enhancers in mouse and human genome. Nucleic Acids Res. 2016;44:D164-71.

19. Gao T, He B, Liu S, Zhu H, Tan K, Qian J. EnhancerAtlas: a resource for enhancer annotation and analysis in 105 human cell/tissue types. Bioinformatics. 2016;32:3543-51.

20. Cao Q, Anyansi C, Hu X, Xu L, Xiong L, Tang W, et al. Reconstruction of enhancer-target networks in 935 samples of human primary cells, tissues and cell lines. Nat Genet. 2017;49:1428-36.

21. Javierre BM, Burren OS, Wilder SP, Kreuzhuber R, Hill SM, Sewitz S, et al. Lineage-specific genome architecture links enhancers and non-coding disease variants to target gene promoters. Cell. 2016;167:1369-84.e19.

22. Ernst J, Kellis M. ChromHMM: automating chromatin-state discovery and characterization. Nat Methods. 2012;9:215-6.

23. Ben-Ari Fuchs S, Lieder I, Stelzer G, Mazor Y, Buzhor E, Kaplan S, et al. GeneAnalytics: an integrative gene set analysis tool for next generation sequencing, RNAseq and microarray data. OMICS. 2016;20:139-51.

**Supplementary Table 1** Basic clinical and genetic data on the 20 pediatric AML cases analyzed by WGS

| Case | Sex | Age | Karyotype | Fusion gene^b^ | SNP-A | Total number of WGS-identified | | |
| --- | --- | --- | --- | --- | --- | --- | --- | --- |
| No.^a^ |  | (years) |  |  | data | SNVs/ | Gene | CNAs |
|  |  |  |  |  | available | indels | variants |  |
| 1 | M | 9 | 46,XY,t(3;21)(q26;q22) | *RUNX1-MECOM* | Yes | 508 | 9 | 0 |
| 2 | M | 8 | 45,X,-Y,t(8;21)(q22;q22) | *RUNX1-RUNXT1* | No | 489 | 5 | 2 |
| 3 | F | 1 | 46,XX,t(17;19)(q21;p13) | ***TCF3-HOXB9*** | Yes | 390 | 5 | 1 |
| 4 | M | 13 | 49,XY,+8,+20,+21/50,idem,+18/51,idem,+18,+18/51,idem, | *KMTA-MLLT10*^c^ | Yes | 667 | 8 | 8 |
|  |  |  | +6,+18/52,idem,+6,+18,+18/52,idem,+6,+i(18)(q10) |  |  |  |  |  |
| 10 | M | 3 | 46,XY,t(2;7)(p15;p22) | *XPO1-TNRC18*^d^ | Yes | 481 | 7 | 0 |
| 14 | M | 16 | 91-94,XXYY,-4,-4,der(5)del(5)(p11)add(5)(q31)x2,-7, | ***PLEKHA5-*** | Yes | 1198 | 18 | 5 |
|  |  |  | +?add(10)(q25),add(12)(q?15),-18,inc | ***ADAMTS20*** |  |  |  |  |
| 15 | F | 0 | 46,XX,t(8;16)(p11;p13) | *KAT6A-CREBP* | Yes | 73 | 0 | 0 |
| 16 | F | 8 | 46,XX,t(8;21)(q22;q22) | *RUNX1-RUNXT1* | Yes | 615 | 6 | 0 |
| 18 | F | 6 | 46,XX,t(8;21)(q22;q22) | *RUNX1-RUNXT1* | Yes | 959 | 11 | 0 |
| 21 | M | 7 | 46,XY,t(7;21)(p22;q22) | *RUNX1-USP42* | Yes | 294 | 4 | 2 |
| 22 | F | 12 | 46,XX,t(8;21)(q22;q22)/45,idem,-X | *RUNX1-RUNXT1* | No | 1101 | 8 | 1 |
| 23 | M | 15 | 46,XY,t(6;9)(p22;q34) | *DEK-NUP214* | Yes | 504 | 2 | 0 |
| 24 | F | 2 | 46,XX,der(5)t(5;10)(p13;q22),der(10)t(10;17)(q22;?),der(10) | *PML-RARA* | No | 143 | 0 | 2 |
|  |  |  | t(10;17)(q24;?),der(15)t(5;15)(p13;q24),der(17)t(15;17) | ***RAP11FIP2-NEURL4*** |  |  |  |  |
|  |  |  | (q24;q21)t(15;17)(q?;p?)t(15;17)(q24;q21)t(10;17)(q24;p?) |  |  |  |  |  |
| 7 | F | 17 | 46,XX | No | Yes | 898 | 8 | 0 |
| 8 | M | 8 | 46,XY,del(7)(q22)/45-47,idem,+1-2mar | No | Yes | 637 | 6 | 3 |
| 9 | F | 12 | 46,XX | No | Yes | 335 | 4 | 0 |
| 11 | M | 3 | 47,XY,der(7)t(1;7)(q24;p21),+21 | No | Yes | 277 | 1 | 3 |
| 12 | F | 14 | 46,XX,del(9)(q11),der(11)del(11)(q13q21)del(11)(q24) | No | Yes | 618 | 9 | 2 |
| 13 | F | 2 | 46,XX,r(7),der(17)t(7;17)(q?;p13)/46,idem,del(6)(q15q23)/ | No | Yes | 500 | 9 | 2 |
|  |  |  | 47,idem,+10 |  |  |  |  |  |
| 19 | M | 2 | 46,XY,t(11;12)(q13;p13),del(13)(q?14q?22)/47,idem, | No | No | 233 | 3 | 3 |
|  |  |  | del(3)(q25),+21/47,idem,t(3;14)(q26;q21),+21/50,idem, |  |  |  |  |  |
|  |  |  | t(3;14),+4,+6,+8,+21 |  |  |  |  |  |

^a^Cases with WGS-identified gene fusions are listed before the cases without fusions. Initially, 25 AML cases were included in the analysis. Five of these (#5, 6, 17, 20, and 25) failed the quality controls prior to, or after, sequencing; however, the original case numbers were kept.

^b^Novel fusion genes are indicated in bold type.

^c^This fusion was not identified in clinical routine diagnostics.

^d^Novel fusion in AML but has previously been reported in one case of acute lymphoblastic leukemia [1]. Lack of RNA precluded RT-PCR verification of this fusion in our case; however, the breakpoints in the cytogenetically identified t(2;7)(p15;p22) supported the presence of the fusion.

AML, acute myeloid leukemia; CNAs, copy number abnormalities; F, female; M, male; SNP-A, single nucleotide polymorphism array; SNVs, single nucleotide variants; WGS, whole genome sequencing.

**REFERENCE**

1. Gu Z, Churchman M, Roberts K, Li Y, Liu Y, Harvey RC, et al. Genomic analyses identify recurrent *MEF2D* fusions in acute lymphoblastic leukaemia. Nat Commun. 2016;7:13331.

**Supplementary Table 2** The three novel in-frame fusion genes in pediatric AML identified by WGS and verified by RT-PCR

| Gene fusion | Names,^b^ functions,^c^ and expression patterns^d^ of the | Chromosome | Breakpoint | Fusion partners in other neoplastic disorders |
| --- | --- | --- | --- | --- |
| (software)^a^ | fusion gene partners | band^e^ | position^e^ | (types)^f^ |
| *PLEKHA5*-*ADAMTS20* | PLEKHA5: pleckstrin homology domain containing | 12p12.3 | 12:19452636 | *ACRBP* (PaC), *ACTG1* (LC), *DCTN2* (SCC), *AEBP2* |
| (DELLY) | A5. A protease cleaved and secreted to extracellular |  |  | (GC), *ALB* (HCC), *APLP2* (RCC), *CASC1* (LC), |
|  | matrix. Expressed in multiple normal tissues, *e.g.*, |  |  | *COX7C* (MalM), *CPE* (AC), *CTTN* (BrC), *DTX3* |
|  | bone marrow, lymph nodes, and white blood cells. |  |  | (AC), *EEF2* (LC), *EPS8* (OC), *FGB* (LC), |
|  | Highly expressed in several neoplasms, such as |  |  | *FGF6* (CC), *GPC6* (BrC), *HSPH1* (SCC), *KRT19* |
|  | carcinoids, colorectal and liver carcinomas, and |  |  | (LC), *NDRG1* (RCC), *P4HB* (BrC), *PIK3C2G* (BlC), |
|  | testis cancer. |  |  | *R3HDM2* (LC), *RASGEF1C* (HCC), *RERGL* (GC), |
|  |  |  |  | *SINHCAF* (EnC), *SLCO1B1* (OC), *ST8SIA1* (BrC), |
|  |  |  |  | *TSC22D1* (LC), *UHRF1* (MesT), *VIM* (MalM) |
|  |  |  |  |  |
|  | ADAMTS20: ADAM metallopeptidase with | 12q12 | 12:43767937 | *C12ORF43* (AC), *CBFA2T2* (ACC), *HOXC4* (ACC), |
|  | thrombospondin type 1 motif 20. A metallopeptidase involved in protein metabolism. |  |  | *MYRFL* (MesT) |
|  | Expressed in, *e.g.*, bone marrow, lymph nodes, |  |  |  |
|  | and thymus. Moderate expression in most |  |  |  |
|  | malignancies, such as glioma and testis cancer. |  |  |  |
|  |  |  |  |  |
| *RAB11FIP2*-*NEURL4* | RAB11FIP2: RAB11 family interacting protein 2. | 10q26.11 | 10:119782909 | None |
| (Manta, novoBreak) | Involved in membrane trafficking. Expressed in, |  |  |  |
|  | *e.g.*, bone marrow, lymph nodes, and white blood |  |  |  |
|  | cells. Expressed in most cancers except lymphomas. |  |  |  |
|  |  |  |  |  |
|  | NEURL4: neuralized E3 ubiquitin protein ligase 4. | 17p13.1 | 17:177220413 | *MSI2* (MalM), *SOX15* (MesT) |
|  | Involved in centriole binding during mitosis. |  |  |  |
|  | Expressed in, *e.g.*, brain, skeletal muscle, and skin. |  |  |  |
|  | Moderately expressed in most cancers. |  |  |  |
|  |  |  |  |  |
| *TCF3*-*HOXB9* | TCF3: transcription factor 3. Binds to E-box | 19p13.3 | 19:1615882 | *ABHD17A* (PrC), *AP3D1* (BrC), *APC2* (PrC), |
| (novoBreak) | sequences. Important for lymphopoiesis. Widely |  |  | *C19orf25* (GC), *DNAJC11* (GC), *FLI1* (ALL), |
|  | expressed in, *e.g.*, in bone marrow, lymph nodes, |  |  | *HLF* (ALL), *MEX3D* (EsC), *NOP2* (ALL), *OAZ1* |
|  | and white blood cells. Expressed in some |  |  | (CLL, MM), *PBX1* (ALL, AML, LC), *PLEKHJ1* |
|  | malignancies, such as carcinoids, colorectal cancer, |  |  | (PrC), *REXO1* (OC), *SYTL5* (BrC), *TEF* (ALL), |
|  | lymphomas, and testis cancer. |  |  | *TFPT* (ALL), *TPM4* (MM), *ZNF384* (ALL) |
|  |  |  |  |  |
|  | HOXB9: homeobox B9. A transcription factor that | 17q21.32 | 17:46701268 | *BCAS3* (BrC), *NIPBL* (AML), *SKAP1* (OC), *USP32* |
|  | binds to Homeobox DNA. Involved in cell |  |  | (LC) |
|  | proliferation and differentiation. Expressed in most |  |  |  |
|  | normal tissues, *e.g.*, bone marrow, lymph nodes, |  |  |  |
|  | thymus, and white blood cells. Highly expressed in |  |  |  |
|  | colorectal cancer. |  |  |  |

AC, astrocytoma; ACC, adenoid cystic carcinoma; ALL, acute lymphoblastic leukemia; AML, acute myeloid leukemia; BlC, bladder cancer; BrC,

breast cancer; CC, colon cancer; CLL, chronic lymphocytic leukemia; EnC, endometrial cancer; EsC, esophageal cancer; GC, gastric cancer; HCC, hepatocellular cancer; LC, lung cancer; MesT, mesenchymal tumor; MalM, malignant melanoma; MM, multiple myeloma; OC, ovarian cancer; PaC, pancreatic cancer; PrC, prostate cancer; RCC, renal cell carcinoma; RT-PCR, reverse transcription polymerase chain reaction; SCC, squamous cell carcinoma; WGS, whole genome sequencing.

^a^Software used to identify the fusion gene.

^b^HUGO Gene Nomenclature Committee (https://www.genenames.org/).

^c^RefSeqGene (https://www.ncbi.nlm.nih.gov/refseq/rsg/) and Online Mendelian Inheritance in Man (https://omim.org).

^d^GeneCards (https://www.genecards.org/) and The Human Protein Atlas (https://www.proteinatlas.org/).

^e^Ensembl, genome assembly GRCh38.p13 [GRCh37 (hg19) elsewhere] (http://www.ensembl.org/index.html).

^f^Mitelman Database of Chromosome Aberrations and Gene Fusions in Cancer (https://mitelmandatabase.isb-cgc.org/).

**Supplementary Table 3** Copy number abnormalities and uniparental isodisomies detected by WGS

| Case | Fusion gene | CNA/ | Size (Mb) of | Cytogenetic | Detected by |
| --- | --- | --- | --- | --- | --- |
| No. |  | UPID | CNA/UPID | correlate | SNP-A |
| 1 | Yes | None | NA | NA | NA |
| 2 | Yes | del(X)(p22.33) | 2.69 | No | Not done |
|  |  | -Y | 57 | Yes | Not done |
| 3 | Yes | del(9)(p21.3p21.3) | 0.29 | No | No |
| 4 | Yes | dup(1)(q21q44) | 125 | No | Yes |
|  |  | +6 | 171 | Yes | Yes |
|  |  | +8 | 145 | Yes | Yes |
|  |  | del(10)(p12.31p12.31) | 0.56 | No | No |
|  |  | del(11)(q23.3q23.3) | 0.25 | No | Yes |
|  |  | +18 | 80 | Yes | Yes |
|  |  | +20 | 64 | Yes | Yes |
|  |  | +21 | 47 | Yes | Yes |
| 10 | Yes | None | NA | NA | NA |
| 14 | Yes | del(5)(q21.3q35.3) | 76 | No | Yes |
|  |  | del(7)(q35q36.2) | 5.98 | No | Yes |
|  |  | UPID(9) | 138 | NA | Yes |
|  |  | +10 | 134 | No | Yes |
|  |  | del(12)(p12.2p12.3) | 0.59 | No | Yes |
|  |  | del(12)(p13.1p13.31) | 3.72 | No | Yes |
|  |  | UPID(15) | 102 | NA | Yes |
| 15 | Yes | None | NA | NA | NA |
| 16 | Yes | None | NA | NA | NA |
| 18 | Yes | None | NA | NA | NA |
| 21 | Yes | del(7)(p22.1p22.3) | 6.2 | No | Yes |
|  |  | del(21)(q22.12q22.3) | 10.55 | No | Yes |
| 22 | Yes | -X | 156 | Yes | Not done |
| 23 | Yes | None | NA | NA | NA |
| 24 | Yes | del(10)(q22.1q22.1) | 0.04 | No | Not done |
|  | Yes | del(17)(q21.1q21.1) | 0.04 | No | Not done |
| 7 | No | None | NA | NA | NA |
| 8 | No | del(7)(q21.2q36.3) | 67 | Yes | Yes |
|  |  | dup(14)(q32.2q32.33) | 7.97 | No | Yes |
|  |  | del(21)(q21.1q21.1) | 0.8 | No | No |
| 9 | No | None | NA | NA | NA |
| 11 | No | dup(1)(q21q44) | 125 | Yes | Yes |
|  |  | del(7)(p21.2p22.3) | 16.3 | Yes | Yes |
|  |  | +21 | 47 | Yes | Yes |
| 12 | No | del(9)(q21.11q33.1) | 51.4 | Yes | Yes |
|  |  | del(11)(q14.1q23.3) | 34.1 | Yes | Yes |
| 13 | No | del(7)(p21.3p22.3) | 10.15 | No | Yes |
|  |  | del(17)(p13.1p13.3) | 7.48 | Yes | Yes |
| 19 | No | del(3)(q13.2q22.1) | 16.92 | Yes | Not done |
|  |  | del(3)(q25.1q25.31) | 5.31 | Yes | Not done |
|  |  | del(13)(q13.3q14.3) | 14.21 | Yes | Not done |

CNA, copy number abnormality; NA, not applicable; SNP-A, single nucleotide polymorphism array; UPID, uniparental

isodisomy; WGS, whole genome sequencing.

**Supplementary Table 4** Median frequencies of the different single base substitutions in cases with and without *RUNX1*-*RUNX1T1* in the present study and in the TCGA dataset

| Single base | Frequencies | | *P*-value^a^ |
| --- | --- | --- | --- |
| substitution | *RUNX1*-*RUNX1T1*- | *RUNX1*-*RUNX1T1*- |  |
|  | positive cases | negative cases |  |
| Present study |  |  |  |
| C>A | 0.247183484 | 0.126650943 | **0.0008256** |
| C>G | 0.052163058 | 0.055474239 | 0.5536 |
| C>T | 0.433905569 | 0.491581219 | 0.09948 |
| T>A | 0.064353538 | 0.079086434 | 0.2114 |
| T>C | 0.138612737 | 0.159638859 | 0.1482 |
| T>G | 0.058164548 | 0.071895651 | 0.3352 |
|  |  |  |  |
| TCGA dataset |  |  |  |
| C>A | 0.151515152 | 0.1 | **0.2316** |
| C>G | 0.031818182 | 0.03125 | 0.8701 |
| C>T | 0.481818182 | 0.5 | 0.916 |
| T>A | 0.1 | 0.083333333 | 0.5574 |
| T>C | 0.062695925 | 0.090909091 | 0.8087 |
| T>G | 0.018181818 | 0 | 0.7303 |

^a^Wilcoxon rank sum test.

**Supplementary Table 5** Frequencies of the different single base substitution mutational signatures in the gene fusion groups in the TCGA dataset

| Mutational | *RUNX1-RUNX1T1* | *CBFB-MYH11* | *PML-RARA* | Other fusion | No fusion |
| --- | --- | --- | --- | --- | --- |
| signature | *(168/6)*^a^ | *(1135/9)*^a^ | *(207/14)*^a^ | (1596/51)^a^ | (5720/117)^a^ |
| SBS1 | 0.17 | 0 | 0.44 | 0.30 | 0.04 |
| SBS2 | 0 | 0 | 0 | 0 | 0 |
| SBS3 | 0 | 0 | 0 | 0 | 0 |
| SBS4 | 0 | 0 | 0.05 | 0.03 | 0 |
| SBS5 | 0 | 0 | 0 | 0 | 0 |
| SBS6 | 0.24 | 0.24 | 0.02 | 0.07 | 0.35 |
| SBS7a | 0.02 | 0.02 | 0 | 0.02 | 0.02 |
| SBS7b | 0.04 | 0.02 | 0 | 0 | 0 |
| SBS7c | 0.01 | 0.01 | 0 | 0 | 0 |
| SBS7d | 0.07 | 0 | 0 | 0 | 0 |
| SBS8 | 0 | 0.04 | 0 | 0 | 0 |
| SBS9 | 0 | 0 | 0 | 0 | 0 |
| SBS10a | 0 | 0 | 0 | 0 | 0 |
| SBS10b | 0.05 | 0.03 | 0 | 0 | 0.01 |
| SBS11 | 0 | 0 | 0 | 0.03 | 0 |
| SBS12 | 0 | 0 | 0 | 0 | 0 |
| SBS13 | 0.03 | 0 | 0 | 0 | 0 |
| SBS14 | 0 | 0 | 0 | 0 | 0 |
| SBS15 | 0 | 0 | 0.14 | 0 | 0 |
| SBS16 | 0 | 0 | 0 | 0.04 | 0 |
| SBS17a | 0.01 | 0.01 | 0 | 0 | 0.01 |
| SBS17b | 0 | 0 | 0 | 0 | 0 |
| **SBS18** | **0.11** | **0** | **0** | **0** | **0** |
| SBS19 | 0 | 0.03 | 0.01 | 0 | 0.02 |
| SBS20 | 0 | 0 | 0 | 0.03 | 0.01 |
| SBS21 | 0.04 | 0 | 0 | 0 | 0 |
| SBS22 | 0 | 0.05 | 0 | 0.02 | 0.05 |
| SBS23 | 0 | 0 | 0 | 0.05 | 0 |
| SBS24 | 0 | 0 | 0 | 0.04 | 0.01 |
| SBS25 | 0 | 0 | 0 | 0 | 0 |
| SBS26 | 0 | 0 | 0 | 0 | 0 |
| SBS28 | 0 | 0 | 0 | 0 | 0 |
| SBS29 | 0.04 | 0 | 0 | 0 | 0 |
| SBS30 | 0 | 0.15 | 0.13 | 0.06 | 0.05 |
| SBS31 | 0.18 | 0 | 0.07 | 0 | 0 |
| SBS32 | 0 | 0 | 0 | 0 | 0 |
| SBS33 | 0 | 0.01 | 0.03 | 0 | 0.01 |
| SBS34 | 0 | 0 | 0 | 0 | 0 |
| SBS35 | 0 | 0 | 0 | 0.02 | 0.06 |
| SBS36 | 0 | 0 | 0 | 0 | 0 |
| SBS37 | 0 | 0 | 0 | 0 | 0 |
| SBS38 | 0 | 0 | 0 | 0 | 0 |
| SBS39 | 0 | 0 | 0.11 | 0.07 | 0.04 |
| SBS40 | 0 | 0 | 0 | 0 | 0 |
| SBS41 | 0 | 0 | 0 | 0 | 0 |
| SBS42 | 0 | 0.16 | 0 | 0.12 | 0.22 |
| SBS44 | 0 | 0.05 | 0 | 0 | 0 |
| SBS84 | 0 | 0.16 | 0 | 0.09 | 0.08 |
| SBS85 | 0 | 0.02 | 0 | 0 | 0.03 |

^a^Numbers of unique single nucleotide variant sites and of cases available in each gene fusion group in

the TCGA dataset (https://www.cbioportal.org/study/summary?id=laml_tcga_pub).

**Supplementary Table 6** The 123 single nucleotide variants and insertions/deletions within coding genes identified by WGS

| Gene^a^ | Case | SNV/indel | Type | Genomic | VAF | Software tools | | Known | Verified |
| --- | --- | --- | --- | --- | --- | --- | --- | --- | --- |
|  | No. |  | of | position | % | SIFT | PolyPhen | variation | yes/no |
|  |  |  | SNV |  |  | (ranking score)^b^ | (ranking score)^b^ | (dbSNPrs/ | (method) |
|  |  |  |  |  |  |  |  | COSMIC) |  |
| ***ADAMTS17*** | 10 | c.3151C>T (p.R1051*) | Nons | 15:100514744 | 36.6 | NA | NA | rs373641684 | Yes (SS) |
| ***AFF1*** | 3 | c.904C>T (p.R302W) | Miss | 4:87968591 | 31.6 | Del (0.01) | Pro dam (1) | COSM1058567 | Yes (WES) |
| ***AGAP1*** | 10 | c.1856G>A (p.R619H) | Miss | 2:236949450 | 23.3 | Del (0.03) | Pos dam (0.893) | rs780244856 | Yes (SS) |
| ***ALDH1B1*** | 18 | c.214G>A (p.D72N) | Miss | 9:38395959 | 31.0 | Tol (0.07) | Pro dam (0.987) | Novel | Yes (SS) |
| ***APLP1*** | 18 | c.1036G>A (p.D346N) | Miss | 19:36364595 | 46.4 | Del (0.01) | Pos dam (0.52) | rs753937698 | Yes (SS) |
| *ARHGAP21* | 1 | c.4964G>A (p.R1655Q) | Miss | 10:24874254 | 56.0 | Tol (0.24) | Ben (0.045) | rs145888629 | No (SS) |
| ***ARID3A*** | 13 | c.706G>C (p.D236H) | Miss | 19:960104 | 41.2 | Del (0) | Pro dam (0.982) | Novel | Yes (SS) |
| ***ASB5*** | 19 | c.406G>A (p.G136S) | Miss | 4:177142730 | 24.3 | Del (0.05) | Pro dam (0.984) | Novel | Yes (SS) |
| ***ASCC3*** | 4 | c.3800A>G (p.Y1267C) | Miss | 6:101090558 | 19.4 | Del (0) | Pos dam (0.904) | Novel | Yes (SS) |
| ***ATP5B*** | 4 | c.841C>T (p.R281W) | Miss | 12:57036567 | 17.9 | Del LC (0.05) | Pro dam (0.999) | Novel | Yes (SS) |
| ***ATP10D*** | 12 | c.2270C>A (p.A757D) | Miss | 4:47560126 | 39.1 | Del (0.01) | Ben (0.071) | Novel | Yes (SS) |
| ***ATRNL1*** | 14 | c.2441G>A (p.R814H) | Miss | 10:117059569 | 27.1 | Tol (0.08) | Pro dam (0.999) | rs781892443, | Yes (SS) |
|  |  |  |  |  |  |  |  | COSM347390 |  |
| ***BCOR*** | 1 | c.2955T>G (p.Y985*) | Nons | X:39931644 | 83.3 | NA | NA | Novel | Yes (DS) |
| ***BCORL1*** | 1 | c.3281_3282insC | FS-ins | X:129150029- | 100 | NA | NA | Novel | Yes (SS) |
|  |  | (p.E1094Dfs*16) |  | 129150030 |  |  |  |  |  |
| ***BCORL1*** | 1 | c.3283_3284insC | FS-ins | X:129150031- | 100 | NA | NA | Novel | Yes (SS) |
|  |  | (p.S1095Tfs*15) |  | 129150032 |  |  |  |  |  |
| *C6orf120* | 14 | c.484G>T (p.G162C) | Miss | 6:170103039 | 66.7 | Tol (0.13) | Ben (0.028) | Novel | Yes (SS) |
| ***CACNA1E*** | 14 | c.238A>T (p.K80*) | Nons | 1:181453118 | 32.1 | NA | NA | Novel | Yes (SS) |
| ***CAMK2G*** | 14 | c.875G>C (p.R292P) | Miss | 10:75602244 | 34.2 | Del LC (0) | Pos dam (0.767) | rs397514627 | Yes (SS) |
| *CCND1* | 22 | c.871_879delCGGGACGTG | IF-del | 11:69466027- | 28.6 | NA | NA | COSM931398, | No (SS) |
|  |  | (p.R291_V293del) |  | 69466035 |  |  |  | COSM1585977 |  |
| ***CDH26*** | 9 | c.2327G>A (p.R776Q) | Miss | 20:58606440 | 42.9 | Del LC (0.03) | Ben (0.238) | Novel | Yes (WES) |
| ***CHD3*** | 14 | c.3683G>C (p.R1228P) | Miss | 17:7806600 | 50.0 | NA | Pos dam (0.767) | Novel | Yes (SS) |
| ***CHD4*** | 1 | c.3644T>G (p.L1215R) | Miss | 12:6696937 | 34.2 | Del (0) | Ben (0.012) | COSM1586694, | Yes (SS) |
|  |  |  |  |  |  |  |  | COSM942709, |  |
|  |  |  |  |  |  |  |  | COSM942708 |  |
| ***CHIA*** | 7 | c.175G>T (p.A59S) | Miss | 1:111854931 | 53.8 | Tol (0.16) | Pro dam (0.992) | rs764153298 | Yes (WES) |
| ***CLCN2*** | 7 | c.1931G>A (p.R644H) | Miss | 3:184071135 | 38.5 | Del (0) | Pro dam (0.932) | COSM1042047 | Yes (WES) |
| *CLDN17* | 16 | c.76C>A (p.L26I) | Miss | 21:31538860 | 51.6 | Tol (1) | Ben (0.058) | COSM1248390 | Yes (SS) |
| ***CLTC*** | 22 | c.4813G>A (p.E1605K) | Miss | 17:57763155 | 14.7 | Del (0) | Pos dam (0.636) | COSM3362258 | Yes (SS) |
| ***CREBBP*** | 22 | c.3234_3238delGCAGC | FS-del | 16:3817733- | 26.5 | NA | NA | Novel | Yes (SS) |
|  |  | (p.Q1079Afs*6) |  | 3817737 |  |  |  |  |  |
| ***CTCF*** | 13 | c.679_694delGATTTTGAGG | FS-del | 16:67645412- | 37.0 | NA | NA | Novel | Yes (DS) |
|  |  | AAGAAC (p.D227Sfs*18) |  | 67645427 |  |  |  |  |  |
| ***DHX15*** | 2 | c.664C>G (p.R222G) | Miss | 4:24572314 | 63.2 | Del (0) | Pro dam (1) | Novel | Yes (SS) |
| ***DNMT3A*** | 9 | c.2645G>A (p.R882H) | Miss | 2:25457242 | 61.9 | Del (0) | Pro dam (0.993) | rs147001633, | Yes (WES) |
|  |  |  |  |  |  |  |  | COSM52944, |  |
|  |  |  |  |  |  |  |  | COSM442676 |  |
| ***ECT2L*** | 10 | c.416G>A (p.G139D) | Miss | 6:139164189 | 29.4 | Del (0) | Pro dam (0.962) | Novel | Yes (SS) |
| ***EPB41L4B*** | 16 | c.2066C>A (p.P689Q) | Miss | 9:111956657 | 38.2 | Del LC (0.01) | Pro dam (0.919) | Novel | Yes (SS) |
| ***FAM179B*** | 14 | c.2676G>C (p.Q892H) | Miss | 14:45475242 | 57.1 | Tol (0.08) | Pos dam (0.819) | Novel | Yes (SS) |
| *FAM184B* | 12 | c.755C>T (p.S252L) | Miss | 4:17710654 | 39.3 | Tol (0.28) | Ben (0.035) | Novel | Yes (SS) |
| *GAD2* | 8 | c.1576C>T (p.L526F) | Miss | 10:26581912 | 61.5 | Del (0) | Pro dam (0.994) | Novel | No (SS) |
| ***GALNT18*** | 10 | c.328C>T (p.R110W) | Miss | 11:11470391 | 51.5 | Del (0) | Pos dam (0.893) | Novel | Yes (SS) |
| ***GATA1*** | 13 | c.721_732delCTCATCCGGC | IF-del | X:48650847- | 27.3 | NA | NA | Novel | Yes (DS) |
|  |  | CC (p.L241_P244del) |  | 48650858 |  |  |  |  |  |
| ***GATA2*** | 12 | c.972G>C (p.K324N) | Miss | 3:128202748 | 51.6 | Del (0) | Pro dam (0.994) | Novel | Yes (DS) |
| ***GJB1*** | 18 | c.220G>T (p.V74L) | Miss | X:70443777 | 44.1 | Del (0.01) | Ben (0.102) | Novel | Yes (SS) |
| ***GRIK2*** | 4 | c.1654G>A (p.V552I) | Miss | 6:102337644 | 29.7 | Del (0.03) | Ben (0.277) | rs780955412, | Yes (SS) |
|  |  |  |  |  |  |  |  | COSM1439428, |  |
|  |  |  |  |  |  |  |  | COSM1439427 |  |
| *H3F3C* | 8 | c.237C>A (p.N79K) | Miss | 12:31944864 | 24.1 | Tol LC (1) | Ben (0) | rs768711923 | No (SS) |
| *H3F3C* | 8 | c.265G>A (p.V89I) | Miss | 12:31944836 | 20 | Tol LC (1) | Ben (0.001) | rs148314204, | No (SS) |
|  |  |  |  |  |  |  |  | COSM693346 |  |
| *H3F3C* | 8 | c.312G>C (p.L104F) | Miss | 12:31944789 | 16.2 | Tol LC (1) | Ben (0) | rs765335762 | No (SS) |
| ***HEATR1*** | 3 | c.2177C>T (p.A726V) | Miss | 1:236748389 | 19.2 | Del (0.01) | Ben (0.081) | Novel | Yes (WES) |
| ***HPCA*** | 22 | c.353G>T (p.R118L) | Miss | 1:33354852 | 25.9 | Del (0.03) | Ben (0.168) | Novel | Yes (SS) |
| ***HSD17B1*** | 7 | c.185C>G (p.T62R) | Miss | 17:40705229 | 43.5 | Del (0.03) | Ben (0.218) | rs61738799 | Yes (WES) |
| ***JAK3*** | 13 | c.1718C>T (p.A573V) | Miss | 19:17948006 | 27.3 | Del (0.05) | Pro dam (0.959) | COSM34215 | Yes (SS) |
| ***KANSL1*** | 13 | c.2158_2159insA | FS-ins | 17:44117112- | 43.3 | NA | NA | Novel | Yes (SS) |
|  |  | (p.R720Qfs*2) |  | 44117112 |  |  |  |  |  |
| ***KIT*** | 16 | c.2466T>G (p.N822K) | Miss | 4:55599340 | 44.8 | Del (0) | Pro dam (1) | COSM1322 | Yes (DS) |
| ***KIT*** | 22 | c.1255_1257delGAC | IF-del | 4:55589771- | 30.8 | NA | NA | Novel | Yes (SS) |
|  |  | (p.D419del) |  | 55589771 |  |  |  |  |  |
| ***KLHL30*** | 10 | c.556G>A (p.D186N) | Miss | 2:239049951 | 37.5 | Del (0.05) | Ben (0.05) | rs778793438 | Yes (SS) |
| ***KMT2C*** | 12 | c.13750C>T (p.R4584W) | Miss | 7:151845262 | 45.5 | NA | Pro dam (1) | rs747850540, | Yes (SS) |
|  |  |  |  |  |  |  |  | COSM1087517, |  |
|  |  |  |  |  |  |  |  | COSM1087516 |  |
| ***KRAS*** | 1 | c.35G>A (p.G12D) | Miss | 12:25398284 | 26.9 | Del (0) | Ben (0.361) | rs121913529, | Yes (DS) |
|  |  |  |  |  |  |  |  | COSM521, |  |
|  |  |  |  |  |  |  |  | COSM1135366 |  |
| ***KRAS*** | 10 | c.38G>A (p.G13D) | Miss | 12:25398281 | 40.0 | Del (0.04) | Pos dam (0.506) | rs112445441, | Yes (DS) |
|  |  |  |  |  |  |  |  | COSM532, |  |
|  |  |  |  |  |  |  |  | COSM1140132 |  |
| *L3MBTL1* | 4 | c.829G>A (p.E277K) | Miss | 20:42157329 | 44.2 | Tol (0.54) | Ben (0.015) | Novel | Yes (SS) |
| ***LARP1*** | 18 | c.1747G>T (p.D583Y) | Miss | 5:154181828 | 53.8 | Del (0) | Pro dam (1) | rs765076218 | Yes (SS) |
| ***LMO2*** | 10 | c.436C>T (p.R146W) | Miss | 11:33886176 | 28.6 | Tol (0.1) | Pro dam (0.995) | Novel | Yes (SS) |
| ***LRRC46*** | 18 | c.271C>T (p.R91C) | Miss | 17:45912764 | 21.9 | Del (0) | Pro dam (0.995) | rs771172378 | Yes (SS) |
| ***MACF1*** | 19 | c.14449C>T (p.R4817*) | Nons | 1:39918368 | 18.9 | NA | NA | COSM345052, | Yes (SS) |
|  |  |  |  |  |  |  |  | COSM345053 |  |
| ***MN1*** | 21 | c.3925_3940delTCCAACAG | FS-del | 22:28146926- | 52.2 | NA | NA | Novel | Yes (SS) |
|  |  | ATTTGGGA (p.S1309Hfs*6) |  | 28146941 |  |  |  |  |  |
| ***MN1*** | 21 | c.3947_3948insA | FS-ins | 22:28146918- | 53.8 | NA | NA | Novel | Yes (SS) |
|  |  | (p.A1317Gfs*35) |  | 28146919 |  |  |  |  |  |
| *MN1* | 21 | c.3942_3944delATT | IF-del | 22:28146922- | 52.0 | NA | NA | Novel | Yes (SS) |
|  |  | (p.F1315del) |  | 28146924 |  |  |  |  |  |
| *MN1* | 22 | c.812C>T (p.P271L) | Miss | 22:28195720 | 48.4 | NA | Ben (0.03) | Novel | Yes (SS) |
| *MT-CYB* | 14 | c.1087C>A (p.L363M) | Miss | MT:15833 | 56.6 | Del LC (0.01) | NA | Novel | No^c^ |
| ***MT-ND1*** | 7 | c.802T>C (p.S268P) | Miss | MT:4108 | 45.4 | Del LC (0.01) | Pro dam (0.984) | Novel | Yes (WES) |
| *MUC7* | 8 | c.517T>C (p.S173P) | Miss | 4:71346978 | 13.9 | Del (0.04) | NA | COSM359922 | No (SS) |
| *MUC17* | 22 | c.5222A>C (p.N1741T) | Miss | 7:100679919 | 13.5 | NA | Ben (0.001) | rs71273403, | Yes (SS) |
|  |  |  |  |  |  |  |  | COSM4161656 |  |
| ***MYLK*** | 23 | c.5446C>T (p.R1816C) | Miss | 3:123337540 | 57.1 | Del (0.01) | Pro dam (0.999) | rs369511530, | Yes (SS) |
|  |  |  |  |  |  |  |  | COSM1632764 |  |
| *MYLK3* | 18 | c.827C>T (p.P276L) | Miss | 16:46771797 | 37.5 | Tol (0.2) | Ben (0.005) | rs762412588 | Yes (SS) |
| ***NCKAP5*** | 2 | c.2280C>A (p.S760R) | Miss | 2:133542104 | 40.0 | Tol (0.31) | Pos dam (0.595) | Novel | Yes (SS) |
| ***NCKAP5L*** | 18 | c.234C>A (p.D78E) | Miss | 12:50195748 | 28.1 | Del (0) | Pro dam (0.998) | Novel | Yes (SS) |
| ***NCOR1*** | 18 | c.1062_1063insG | FS-ins | 17:16049709- | 41.4 | NA | NA | Novel | Yes (SS) |
|  |  | (p.Q355Afs*21) |  | 16049710 |  |  |  |  |  |
| ***NCOR1*** | 18 | c.2080C>T (p.R694*) | Nons | 17:16012202 | 34.8 | NA | NA | COSM1756932 | Yes (SS) |
| *NDN* | 14 | c.509C>T (p.A170V) | Miss | 15:23931856 | 66.7 | Tol (0.06) | Pro dam (0.961) | Novel | No (SS) |
| ***NKAP*** | 13 | c.146G>A (p.R49Q) | Miss | X:119077423 | 42.9 | Tol (0.07) | Pos dam (0.788) | Novel | Yes (SS) |
| ***NOTCH1*** | 14 | c.4793G>C (p.R1598P) | Miss | 9:139399350 | 78.6 | Tol (0.23) | Pos dam (0.897) | COSM13053, | Yes (DS) |
|  |  |  |  |  |  |  |  | COSM305943 |  |
| *NRAS* | 3 | c.182A>G (p.Q61R) | Miss | 1:115256529 | 19.2 | Tol (0.06) | Ben (0.006) | rs11554290, | Yes (WES) |
|  |  |  |  |  |  |  |  | COSM584 |  |
| ***NRAS*** | 4 | c.181C>A (p.Q61K) | Miss | 1:115256530 | 22.2 | Del (0.01) | Ben (0.045) | rs121913254, | Yes (SS) |
|  |  |  |  |  |  |  |  | COSM580 |  |
| *NRAS* | 4 | c.182A>G (p.Q61R) | Miss | 1:115256529 | 16.7 | Tol (0.06) | Ben (0.006) | rs11554290, | Yes (SS) |
|  |  |  |  |  |  |  |  | COSM584 |  |
| ***NRG2*** | 1 | c.1667G>A (p.R556Q) | Miss | 5:139231294 | 66.7 | Del (0) | Pro dam (0.999) | rs370889827 | Yes (SS) |
| ***OR1L3*** | 23 | c.852G>T (p.L284F) | Miss | 9:125438260 | 30.6 | Del (0.01) | Pos dam (0.651) | Novel | Yes (SS) |
| *OR1Q1* | 14 | c.782G>A (p.R261Q) | Miss | 9:125377798 | 50.0 | Tol (0.59) | Ben (0.017) | rs368242898 | Yes (SS) |
| *OR4A15* | 14 | c.46G>T (p.V16F) | Miss | 11:55135405 | 52.0 | NA | Ben (0) | Novel | Yes (SS) |
| *OR8H1* | 12 | c.489C>A (p.S163R) | Miss | 11:56058050 | 39.1 | Tol (0.13) | Ben (0.033) | Novel | No (SS) |
| ***PDLIM5*** | 1 | c.316G>A (p.E106K) | Miss | 4:95497118 | 56.5 | Del (0.04) | Ben (0.087) | Novel | Yes (SS) |
| ***PDZD4*** | 12 | c.68G>A (p.G23E) | Miss | X:153074043 | 46.4 | Del (0) | Pos dam (0.859) | Novel | Yes (SS) |
| *PFAS* | 22 | c.3584G>A (p.R1195H) | Miss | 17:8172052 | 17.1 | Tol (0.12) | Ben (0.155) | rs372608754, | No (SS) |
|  |  |  |  |  |  |  |  | COSM3932792 |  |
| ***PHLDB1*** | 12 | c.2020C>T (p.R674C) | Miss | 11:118502116 | 50.0 | Del (0) | Ben (0) | rs116066979 | Yes (SS) |
| *PIGC* | 14 | c.781A>G (p.I261V) | Miss | 1:172410982 | 45.7 | Tol (0.49) | Ben (0.007) | rs148171978 | Yes (SS) |
| ***PLCE1*** | 18 | c.2932C>A (p.L978I) | Miss | 10:96006214 | 53.3 | Tol (0.15) | Pos dam (0.844) | Novel | Yes (SS) |
| ***PRELID1*** | 2 | c.199G>C (p.E67Q) | Miss | 5:176731732 | 38.1 | Del (0.05) | Ben (0.233) | Novel | Yes (SS) |
| *PRKAR2B* | 9 | c.239C>T (p.P80L) | Miss | 7:106685591 | 15.6 | Tol (0.08) | Ben (0.019) | Novel | Yes (WES) |
| *PTPN5* | 11 | c.1306C>T (p.R436W) | Miss | 11:18754162 | 18.5 | Del (0.02) | Pro dam (0.988) | rs753955534 | No (SS) |
| ***PTPN11*** | 7 | c.1507G>A (p.G503R) | Miss | 12:112926887 | 34.5 | Del (0.03) | Pro dam (1) | rs397507545, | Yes (WES) |
|  |  |  |  |  |  |  |  | COSM14259 |  |
| ***RAB6A*** | 7 | c.610G>C (p.G204R) | Miss | 11:73388960 | 63.0 | Del (0.03) | Ben (0.011) | Novel | Yes (WES) |
| ***RAC2*** | 16 | c.101C>G (p.P34R) | Miss | 22:37637633 | 30.0 | Del LC (0) | Pro dam (1) | Novel | Yes (SS) |
| ***RASL11A*** | 2 | c.433G>A (p.V145M) | Miss | 13:27847335 | 31.8 | Del (0.04) | Pro dam (0.937) | Novel | Yes (SS) |
| *RBM12B-AS1* | 19 | c.158T>G (p.F53C) | Miss | 8:94752638 | 15.4 | NA | Ben (0.006) | Novel | Yes (SS) |
| ***RNF148*** | 14 | c.886C>T (p.P296S) | Miss | 7:122341919 | 45.5 | Del (0) | Pro dam (0.999) | Novel | Yes (SS) |
| *RPH3AL* | 18 | c.815C>T (p.T272M) | Miss | 17:65506 | 52.9 | Tol (0.06) | Ben (0.033) | rs199836603 | Yes (SS) |
| ***RPL10A*** | 1 | c.562A>G (p.N188D) | Miss | 6:35438435 | 50 | Del (0.02) | Pro dam (0.974) | Novel | Yes (SS) |
| ***RTN1*** | 3 | c.1802C>T (p.T601M) | Miss | 14:60074174 | 25 | Del (0.01) | Pro dam (0.998) | Novel | Yes (WES) |
| ***SAGE1*** | 13 | c.2506C>T (p.R836*) | Nons | X:134994097 | 44.8 | NA | NA | rs782625354, | Yes (SS) |
|  |  |  |  |  |  |  |  | COSM1465794, |  |
|  |  |  |  |  |  |  |  | COSM3843660 |  |
| *SCN5A* | 7 | c.5831G>A (p.R1944Q) | Miss | 3:38592032 | 36.0 | Tol (0.17) | Ben (0.002) | rs767089602 | Yes (WES) |
| *SLIT2* | 4 | c.4463G>C (p.G1488A) | Miss | 4:20620505 | 48.6 | Tol (0.16) | Ben (0.003) | Novel | Yes (SS) |
| ***SMC3*** | 13 | c.109G>A (p.G37R) | Miss | 10:112333482 | 35 | Del (0) | Pro dam (1) | Novel | Yes (DS) |
| *SPATA31A1* | 8 | c.3364G>A (p.E1122K) | Miss | 9:39361126 | 52.9 | Tol (0.44) | Ben (0.227) | Novel | No (SS) |
| ***SYCP2*** | 16 | c.1763C>A (p.S588*) | Nons | 20:58468227 | 42.9 | NA | NA | Novel | Yes (SS) |
| *TEDC2* | 14 | c.368C>T (p.P123L) | Miss | 16:2510988 | 58.1 | Tol (0.08) | Ben (0.007) | rs553437811 | Yes (SS) |
| ***TM9SF4*** | 14 | c.1124G>A (p.R375Q) | Miss | 20:30738461 | 53.6 | Del (0.03) | Pro dam (0.924) | Novel | Yes (SS) |
| ***TMED6*** | 4 | c.307C>T (p.R103W) | Miss | 16:69383461 | 34.4 | Del (0.01) | Pro dam (0.998) | rs755352517 | Yes (SS) |
| ***TMEM132E*** | 14 | c.2375G>T (p.R792L) | Miss | 17:32964671 | 76.2 | Del (0) | Pro dam (0.999) | Novel | Yes (SS) |
| *TTC28* | 16 | c.3788A>G (p.H1263R) | Miss | 22:28490212 | 52.6 | Tol (0.34) | Ben (0.04) | Novel | Yes (SS) |
| ***UBA2*** | 3 | c.319C>T (p.R107*) | Nons | 19:34924278 | 37.5 | NA | NA | COSM214321 | Yes (WES) |
| ***UBE2D2*** | 13 | c.43C>T (p.R15W) | Miss | 5:138979975 | 31.6 | Del LC (0.03) | Ben (0.018) | Novel | Yes (SS) |
| *UNC5D* | 12 | c.2062G>A (p.V688M) | Miss | 8:35608226 | 37.5 | Tol (0.1) | Ben (0.047) | Novel | Yes (SS) |
| ***USF2*** | 9 | c.562C>T (p.Q188*) | Nons | 19:35761482 | 57.7 | NA | NA | Novel | Yes (WES) |
| ***WT1*** | 7 | c.1288C>T (p.R430*) | Nons | 11:32414263 | 65.2 | NA | NA | rs121907906, | Yes (WES) |
|  |  |  |  |  |  |  |  | COSM21401 |  |
| ***ZC3H12A*** | 12 | c.773G>A (p.R258H) | Miss | 1:37947391 | 58.3 | Del (0.03) | Ben (0.411) | rs142695824 | Yes (SS) |
| ***ZFAT*** | 2 | c.131delA (p.E44Gfs*9) | FS-del | 8:135669869 | 48.1 | NA | NA | Novel | Yes (SS) |
| ***ZNF251*** | 14 | c.1441C>T (p.R481*) | Nons | 8:145947604 | 55.6 | NA | NA | rs754338749 | Yes (SS) |
| ***ZNF365*** | 14 | c.676G>A (p.V226M) | Miss | 10:64136628 | 30.6 | Tol (0.11) | Pro dam (0.999) | rs758463434 | Yes (SS) |
| ***ZNF544*** | 21 | c.226G>A (p.E76K) | Miss | 19:58758142 | 34.6 | Del (0.01) | Ben (0.019) | Novel | Yes (SS) |

^a^Bold type indicates genes with variants considered pathogenic, either by resulting in truncation or by being classified as such by SIFT and/or PolyPhen. Bold type and underlined indicates genes previously not implicated in AML.

^b^The ranking scores provided by SIFT and PolyPhen are probability scores that predict the pathogenicity of the variant. For SIFT, a variant is predicted to be damaging if the score is ≤ 0.05 and tolerated if the score is > 0.05. For Polyphen, variants with scores of 0.0 are predicted to be benign and values approaching 1.0 are increasingly confidently predicted to be deleterious. Truncating variants are not scored by SIFT or PolyPhen.

^c^Sanger sequencing could not be performed due to technical reasons.

Ben, benign; dbSNPrs, single nucleotide polymorphism database reference number; Del, deleterious; Del LC, deleterious with low confidence; DS, deep sequencing; FS-del, frameshift deletion; FS-ins, frameshift insertion; IF-del, in-frame deletion; indel, insertion/deletion; Miss, missense; MT, mitochondrial; NA, not applicable; Nons, nonsense; Pos dam, possibly damaging; Pro dam; probably damaging; SNV, single nucleotide variant; SS, Sanger sequencing; Tol, tolerated; Tol LC, tolerated with low confidence; Unk, unknown; VAF, variant allele frequency; WES, whole exome sequencing; WGS, whole genome sequencing.

**Supplementary Table 7** The 133 single nucleotide variants and insertions/deletions in regulatory elements identified by WGS

| Regulatory | SNV/indel | SNV/indel | Case | Target genes/non-coding RNAs listed in at least two datasets^b^ | Dataset^c^ |
| --- | --- | --- | --- | --- | --- |
| element | start site |  |  |  |  |
| (function)^a^ | (region) |  |  |  |  |
| Enhancer (4) | 1:1795013 | A>ACCACCTTCTA | 23 | *CDK11A, CPSF3L, GNB1, MRPL20, NADK, RP1-140A9.1, RP11-54O7.14,* | dbSUPER, EnhancerAtlas |
|  | (intron) | CATGCTGCCTCT |  | *SLC35E2, SSU72* |  |
| Enhancer (4) | 1:27071277 | G>GCCCTCC | 23 | *ARID1A, C1orf172, FGR, GPATCH3, HMGN2, PAFAH2, PIGV*, *RN7SL490P,* | dbSUPER, EnhancerAtlas |
|  | (intron) |  |  | *RN7SL501P, RP5-968P14.2, RPS6KA1, STMN1, ZNF683* |  |
| Enhancer (5) | 1:89776693 | T>G | 22 | *GBP2, GBP5, RP4-620F22.2* | dbSUPER, EnhancerAtlas |
|  | (intergenic) |  |  |  |  |
| Enhancer (4) | 1:117047239 | CT>C | 22 | *ATP1A1, ATP1A1OS, C1orf137, CD2, CD58, IGSF3, PTGFRN, RP5-1086K13.1,* | dbSUPER, EnhancerAtlas, |
|  | (intergenic) |  |  | *SLC22A15* | PCHi-C |
| Enhancer (4) | 1:143992306 | C>A | 21 | *FAM72D, PDE4DIP* | dbSUPER, |
|  | (intron) |  |  |  | EnhancerAtlas |
| Enhancer (7) | 1:167773846 | C>A | 7 | *CREG1, DCAF6, MPC2, MPZL1, POU2F1, RCSD1, RP1-313L4.3, RP1-313L4.4* | EnhancerAtlas, PCHi-C |
|  | (3'Flank) |  |  |  |  |
| Enhancer (4) | 2:37892947 | A>C | 18 | *AC006369.2, ATL2, CDC42EP3, CEBPZ, QPCT, RMDN2, STRN* | dbSUPER, EnhancerAtlas, |
|  | (intron) |  |  |  | PCHi-C |
| Enhancer (7) | 2:68996444 | A>G | 23 | *ARHGAP25, FBXO48, RP11-427H3.3, WDR92* | dbSUPER, EnhancerAtlas |
|  | (intron) |  |  |  |  |
| Enhancer (4) | 2:86080048 | T>A | 3 | *CD8A, RNU6-640P, ST3GAL5, VAMP8* | dbSUPER, EnhancerAtlas |
|  | (intron) |  |  |  |  |
| Enhancer (6) | 4:2264852 | A>C | 2 | *MIR4800, MXD4* | dbSUPER, PCHi-C |
|  | (3'Flank) |  |  |  |  |
| Enhancer (7) | 4:37759913 | G>A | 16 | *PGM2, PTTG2, RELL1, RP11-617D20.1, TBC1D1* | dbSUPER, EnhancerAtlas, |
|  | (intergenic) |  |  |  | PCHi-C |
| Enhancer (4) | 5:126129185 | G>A | 2 | *C5orf63, LMNB1, PHAX* | dbSUPER, EnhancerAtlas |
|  | (intron) |  |  |  |  |
| Enhancer (5) | 5:169706945 | T>G | 23 | *C5orf58, LCP2* | dbSUPER, EnhancerAtlas |
|  | (intron) |  |  |  |  |
| Enhancer (4) | 6:13434471 | A>G | 21 | *CD83, GFOD1, MCUR1, NOL7, RANBP9, SIRT5, TBC1D7* | dbSUPER, EnhancerAtlas |
|  | (intron) |  |  |  |  |
| Enhancer (4) | 6:13434473 | G>A | 21 | *CD83, GFOD1, MCUR1, NOL7, RANBP9, SIRT5, TBC1D7* | dbSUPER, EnhancerAtlas |
|  | (intron) |  |  |  |  |
| Enhancer (5) | 6:15280861 | T>C | 2 | *JARID2* | dbSUPER, EnhancerAtlas |
|  | (intron) |  |  |  |  |
| Enhancer (5) | 7:47537525 | CTCA>C | 1 | *HUS1, LINC00525, TNS3* | dbSUPER, EnhancerAtlas |
|  | (intron) |  |  |  |  |
| Enhancer (7) | 7:69119310 | C>T | 16 | *AUTS2* | dbSUPER, EnhancerAtlas |
|  | (intron) |  |  |  |  |
| Enhancer (5) | 7:92399252 | G>T | 13 | *CCDC132, CDK6, CYP51A1, FAM133B, GATAD1, MTERF, SAMD9, SAMD9L* | dbSUPER, EnhancerAtlas |
|  | (intron) |  |  |  |  |
| Enhancer (4) | 8:38820798 | A>T | 23 | *ADAM9, ASH2L, DDHD2, FGFR1, HTRA4, PLEKHA2, TACC1, TM2D2* | dbSUPER, EnhancerAtlas |
|  | (intron) |  |  |  |  |
| Enhancer (4) | 8:81015443 | T>A | 18 | *MRPS28, RP11-92K15.1, STMN2, TPD52* | dbSUPER, EnhancerAtlas |
|  | (intron) |  |  |  |  |
| Enhancer (7) | 8:135727020 | G>A | 3 | *ZFAT* | dbSUPER, JEME, PCHi-C |
|  | (5'Flank) |  |  |  |  |
| Enhancer (7) | 9:74516055 | G>A | 18 | *ABHD17B, C9orf85, GDA,* ***Y_RNA*** | dbSUPER, EnhancerAtlas |
|  | (intron) |  |  |  |  |
| Enhancer (5) | 9:95796063 | A>G | 14 | *FAM120AOS, FGD3, IARS, SUSD3* | dbSUPER, EnhancerAtlas |
|  | (intron) |  |  |  |  |
| Enhancer (5) | 9:135935879 | C>T | 22 | *CEL, GTF3C5* | dbSUPER, PCHi-C |
|  | (3'Flank) |  |  |  |  |
| Enhancer (4) | 10:96990678 | G>A | 12 | *ALDH18A1, C10orf129, ENTPD1, HELLS, NOC3L, PDLIM1* | dbSUPER, EnhancerAtlas |
|  | (3'Flank) |  |  |  |  |
| Enhancer (7) | 11:33943461 | T>G | 16 | *CAPRIN1, CAT, CSTF3, FBXO3, LMO2, NAT10* | dbSUPER, EnhancerAtlas |
|  | (intergenic) |  |  |  |  |
| Enhancer (5) | 11:65275588 | A>G | 1 | *LTBP3, MALAT1* | dbSUPER, EnhancerAtlas |
|  | (3'Flank) |  |  |  |  |
| Enhancer (5) | 11:128606068 | A>C | 1 | *TP53AIP1* | dbSUPER, EnhancerAtlas, |
|  | (intron) |  |  |  | JEME |
| Enhancer (4) | 12:4381795 | G>A | 7 | *CCND2, CCND2-AS1, CCND2-AS2* | dbSUPER, PCHi-C |
|  | (5'Flank) |  |  |  |  |
| Enhancer (4) | 12:4676409 | T>C | 16 | *C12orf4, RAD51AP1, RP11-234B24.6, RP11-320N7.1* | dbSUPER, EnhancerAtlas |
|  | (intron) |  |  |  |  |
| Enhancer (4) | 12:7066604 | A>ACGAGGTCC | 8 | *C12orf57, CDCA3, COPS7A,* ***EMG1****, ENO2, LEPREL2, LRRC23, MIR141,* | dbSUPER, |
|  | (intron) |  |  | *MIR200C, NOP2, PEX5, PHB2, PTMS, PTPN6, RN7SL380P, SCARNA12,* | EnhancerAtlas, PCHi-C |
|  |  |  |  | *SPSB2, U47924.27, U47924.29, ZNF384* |  |
| Enhancer (6) | 12:57424974 | G>T | 22 | *AC023237.1, ARHGAP9, ATP5B, MIR616, MYL6, MYO1A, PAN2, SHMT2,* | dbSUPER, EnhancerAtlas, |
|  | (intron) |  |  | *STAC3, STAT6, TAC3, ZBTB39* | PCHi-C |
| Enhancer (6) | 12:80323151 | A>G | 1 | *PAWR, PPP1R12A, RP11-84G21.1, RP11-530C5.1, SYT1* | EnhancerAtlas, PCHi-C |
|  | (intron) |  |  |  |  |
| Enhancer (4) | 14:74210765 | AAT>A | 2 | *ACOT1, ACOT2, DNAL1, ELMSAN1, ENTPD5, MIR4505, NUMB, PAPLN,* | dbSUPER, EnhancerAtlas |
|  | (intron) |  |  | *PNMA1, PTGR2, RP3-414A15.10, RP4-693M11.3, RP5-1021I20.1, ZFYVE1,* |  |
|  |  |  |  | *ZNF410* |  |
| Enhancer (6) | 14:78083072 | G>A | 16 | *SPTLC2* | dbSUPER, PCHi-C |
|  | (5'UTR) |  |  |  |  |
| Enhancer (7) | 15:64151392 | C>T | 14 | *DAPK2, FAM96A, HERC1, KIAA0101, LACTB, PIF1, RP11-111E14.1, TPM1* | dbSUPER, EnhancerAtlas |
|  | (intergenic) |  |  |  |  |
| Enhancer (4) | 15:75069819 | G>T | 22 | *ARID3B, CLK3, COX5A, CPLX3, CSK, CYP1A1, CYP1A2, EDC3, FAM219B,* | dbSUPER, JEME |
|  | (5'Flank) |  |  | *ISLR2, LMAN1L, MIR4513, MPI, PPCDC, PTPN9, RPP25, SCAMP2, ULK3* |  |
| Enhancer (5) | 16:4666534 | G>T | 1 | *MGRN1, UBALD1* | dbSUPER, PCHi-C |
|  | (5'Flank) |  |  |  |  |
| Enhancer (7) | 16:11334765 | G>C | 15 | *ATF7IP2, HNRNPCP4, RMI2, SOCS1* | dbSUPER, EnhancerAtlas, |
|  | (5'Flank) |  |  |  | PCHi-C |
| Enhancer (4) | 16:21371969 | G>A | 2 | *CRYM, CTB-31N19.3, DCUN1D3, IGSF6, METTL9, NPIPB3, RNU6-1005P,* | dbSUPER, EnhancerAtlas, |
|  | (intergenic) |  |  | *SNX29P1, ZP2* | JEME |
| Enhancer (6) | 16:50099867 | A>C | 19 | *ADCY7, CNEP1R1, HEATR3, PAPD5, RP11-429P3.3* | JEME, PCHi-C |
|  | (5'UTR) |  |  |  |  |
| Enhancer (4) | 17:20552666 | G>A | 2 | *LGALS9B, UPF3AP2* | EnhancerAtlas, JEME |
|  | (intron) |  |  |  |  |
| Enhancer (4) | 17:20552724 | T>G | 2 | *LGALS9B, UPF3AP2* | EnhancerAtlas, JEME |
|  | (intron) |  |  |  |  |
| Enhancer (7) | 17:55567453 | GCAAGCTCTTTAG | 14 | *CUEDC1, DGKE, MSI2* | dbSUPER, EnhancerAtlas |
|  | (intron) | TCTC>G |  |  |  |
| Enhancer (4) | 17:57926222 | C>T | 7 | *CLTC, DHX40, HEATR6, MIR21, PRR11, PTRH2, RNU6-450P, RPS6KB1,* | dbSUPER, EnhancerAtlas |
|  | (5'Flank) |  |  | *SCARNA20, TRIM37, TUBD1, VMP1* |  |
| Enhancer (5) | 18:60767554 | G>C | 7 | *BCL2, KDSR, RP11-173A16.1, RP11-299P2.1, SERPINB12* | dbSUPER, EnhancerAtlas |
|  | (intergenic) |  |  |  |  |
| Enhancer (7) | 19:1923736 | AGCCTCCCGAGT | 13 | *ABCA7, ABHD17A, AC005943.4, AP3D1, ATP5D, BTBD2, CIRBP, CNN2,* | dbSUPER, EnhancerAtlas |
|  | (intron) | AGCTGGGACTAC |  | *CSNK1G2, CTB-25B13.12, CTB-31O20.9, GADD45B, HMHA1, JSRP1,* |  |
|  |  | AGGCGCCCGCTA |  | *LMNB2, MBD3, NDUFS7, RPS15, SBNO2, STK11* |  |
|  |  | CCGCACCTGGCT |  |  |  |
|  |  | AATTATTTTGTTT |  |  |  |
|  |  | TTGTATTTTTGGT |  |  |  |
|  |  | AGAGACAGGTTT |  |  |  |
|  |  | TCACCGTGTTAGC |  |  |  |
|  |  | CAGGATGGTCTC |  |  |  |
|  |  | AATCTCCTGACCT |  |  |  |
|  |  | CGTGATCCTCCCG |  |  |  |
|  |  | CCTCG>A |  |  |  |
| Enhancer (4) | 19:12894240 | G>T | 18 | *C19orf43, CTD-2659N19.2, CTD-2659N19.10, CTD-2659N19.9, HOOK2, JUNB,* | dbSUPER, EnhancerAtlas, |
|  | (3'Flank) |  |  | *MIR5684, RNASEH2A, PRDX2* | PCHi-C |
| Enhancer (5) | 19:16245605 | G>C | 7 | *HSH2D, RAB8A* | dbSUPER, EnhancerAtlas |
|  | (3'Flank) |  |  |  |  |
| Enhancer (4) | 19:17634178 | C>T | 8 | *FAM129C* | dbSUPER, PCHi-C |
|  | (5'UTR) |  |  |  |  |
| Enhancer (7) | 19:54776313 | C>T | 11 | *LILRB2, LILRB5, MIR4752* | dbSUPER, PCHi-C |
|  | (3'Flank) |  |  |  |  |
| Enhancer (4) | 22:20866142 | T>C | 11 | *AC007731.1, KLHL22, MED15* | dbSUPER, EnhancerAtlas |
|  | (intron) |  |  |  |  |
| Enhancer (4) | 22:23479977 | C>G | 19 | *AC000029.1, BCR, D87024.1, IGLC1, IGLC2, IGLC3, IGLC7,* | dbSUPER, EnhancerAtlas, |
|  | (intron) |  |  | *IGLJ2, IGLJ3, IGLJ7, IGLV1-36, IGLV1-51, IGLV2-8, IGLV3-1,* | PCHi-C |
|  |  |  |  | *IGLV3-16, IGLV4-60, IGLV6-57, IGLV7-43, IGLV9-49, RTDR1,* |  |
|  |  |  |  | *SMARCB1* |  |
| Enhancer (4) | 22:45630714 | T>G | 10 | *CTA-217C2.1, FAM118A, FBLN1, KIAA0930, NUP50, RP1-102D24.5* | dbSUPER, EnhancerAtlas |
|  | (intron) |  |  |  |  |
| Promoter (1) | 1:148248035 | C>A | 22 | *NBPF15, NBPF20, PPIAL4D, RNVU1-14* | EnhancerAtlas, JEME |
|  | (3'Flank) |  |  |  |  |
| Promoter (1) | 1:203734482 | C>A | 24 | *LAX1* | dbSUPER, PCHi-C |
|  | (5'UTR) |  |  |  |  |
| Promoter (1) | 1:212740492 | T>C | 11 | *ATF3, BATF3, RP11-61J19.4, RP11-384C4.7, SNORA16B, TATDN3* | dbSUPER, EnhancerAtlas, |
|  | (intron) |  |  |  | PCHi-C |
| Promoter (1) | 2:3522942 | A>C | 3 | *AC108488.4, AC142528.1, ADI1, COLEC11, RPS7, TRAPPC12, TSSC1-IT1* | EnhancerAtlas, PCHi-C |
|  | (intron) |  |  |  |  |
| Promoter (1) | 2:55644289 | T>C | 23 | *CCDC88A, CCDC104, MTIF2, PRORSD1P, RPS27A, SNORA12* | dbSUPER, EnhancerAtlas, |
|  | (intron) |  |  |  | PCHi-C |
| Promoter (1) | 2:179388618 | C>T | 1 | *DFNB59, FKBP7, PLEKHA3, PRKRA* | EnhancerAtlas, JEME |
|  | (3'Flank) |  |  |  |  |
| Promoter (1) | 4:99849721 | C>A | 22 | *AC019131.1, ADH4, ADH5, EIF4E, METAP1, MIR3684, RAP1GDS1,* | EnhancerAtlas, PCHi-C |
|  | (intron) |  |  | *RP11-571L19.7, TSPAN5* |  |
| Promoter (2) | 6:52860572 | A>T | 8 | *FBXO9, GCM1, GSTA1, GSTA2, GSTA3, GSTA4, GSTA5, ICK, RN7SK,* | dbSUPER, JEME, PCHi-C |
|  | (5'Flank) |  |  | *TMEM14A* |  |
| Promoter (2) | 7:20478487 | A>C | 7 | *ABCB5, ITGB8, MACC1, TWISTNB* | EnhancerAtlas, JEME |
|  | (intergenic) |  |  |  |  |
| Promoter (1) | 8:91013928 | T>G | 24 | *DECR1, NBN, OSGIN2* | dbSUPER, EnhancerAtlas, |
|  | (intron) |  |  |  | PCHi-C |
| Promoter (2) | 9:27528208 | C>T | 14 | *C9orf72, IFNK, MOB3B* | dbSUPER, EnhancerAtlas, |
|  | (intron) |  |  |  | PCHi-C |
| Promoter (2) | 9:93604255 | G>A | 9 | *NFIL3, SYK* | dbSUPER, EnhancerAtlas |
|  | (intron) |  |  |  |  |
| Promoter (1) | 10:73610118 | C>T | 18 | *C10orf54, DDIT4, DNAJB12, PSAP, RP11-472G21.2, SNORA36* | dbSUPER, EnhancerAtlas, |
|  | (intron) |  |  |  | PCHi-C |
| Promoter (1) | 10:101380552 | C>T | 22 | *RP11-85A1.3, SLC25A28* | dbSUPER, PCHi-C |
|  | (5'Flank) |  |  |  |  |
| Promoter (1) | 10:135208146 | C>T | 10 | *ADAM8, CALY, CYP2E1, FUOM, GPR123-AS1, INPP5A, MTG1, PAOX,* | EnhancerAtlas, PCHi-C |
|  | (intron) |  |  | *RP11-108K14.8, TUBGCP2* |  |
| Promoter (1) | 12:29533541 | T>C | 15 | *ERGIC2, FAR2, OVCH1-AS1* | EnhancerAtlas, PCHi-C |
|  | (intron) |  |  |  |  |
| Promoter (1) | 13:113346168 | C>T | 23 | *ATP11A, MCF2L, TUBGCP3* | dbSUPER, EnhancerAtlas, |
|  | (intron) |  |  |  | PCHi-C |
| Promoter (3) | 14:24868107 | G>C | 16 | *NFATC4, NYNRIN* | dbSUPER, PCHi-C |
|  | (5'UTR) |  |  |  |  |
| Promoter (2) | 14:58763288 | C>A | 8 | *ARID4A, C14orf37, FLJ31306, RP11-349A22.5* | dbSUPER, PCHi-C |
|  | (5'Flank) |  |  |  |  |
| Promoter (1) | 14:69258299 | A>C | 19 | *DCAF5, ZFP36L1* | dbSUPER, EnhancerAtlas, |
|  | (intron) |  |  |  | PCHi-C |
| Promoter (1) | 15:31621684 | A>G | 24 | *KLF13* | dbSUPER, |
|  | (intron) |  |  |  | EnhancerAtlas, PCHi-C |
| Promoter (1) | 17:5137621 | A>C | 11 | *RABEP1, RP5-1050D4.3, RP11-333E1.2, SCIMP, SPAG7, ZMYND15, ZNF232* | dbSUPER, EnhancerAtlas |
|  | (intron) |  |  |  |  |
| Promoter (2) | 17:43237074 | G>A | 13 | *AC002117.1, HEXIM2, RP13-890H12.2* | dbSUPER, PCHi-C |
|  | (5'Flank) |  |  |  |  |
| Promoter (2) | 17:47840756 | A>C | 1 | *FAM117A, KAT7, NGFR, RP11-1079K10.2, SLC35B1, SPOP* | dbSUPER, EnhancerAtlas |
|  | (intron) |  |  |  |  |
| Promoter (1) | 17:56408960 | T>C | 12 | *BZRAP1-AS1, MIR142, MIR4736, SUPT4H1* | dbSUPER, PCHi-C |
|  | (5'Flank) |  |  |  |  |
| Promoter (2) | 18:8704911 | G>T | 16 | *ANKRD12, PTPRM, RAB12, RP11-674N23.1, SOGA2, TWSG1, U3* | dbSUPER, EnhancerAtlas |
|  | (intron) |  |  |  |  |
| Promoter (1) | 19:41770323 | C>T | 18 | *CCDC97, HNRNPUL1* | dbSUPER, PCHi-C |
|  | (5'UTR) |  |  |  |  |
| Promoter (1) | 19:55851647 | T>C | 18 | *AC020922.1, BRSK1, COX6B2, CTD-2105E13.6, DNAAF3, IL11, NAT14, RPL28,* | dbSUPER, EnhancerAtlas, |
|  | (intron) |  |  | *SBK2, SUV420H2, TMEM150B* | PCHi-C |
| Promoter (1) | 20:17948810 | C>T | 2 | *MGME1, SNORD17, SNX5* | dbSUPER, PCHi-C |
|  | (intron) |  |  |  |  |
| Promoter (1) | 21:45579918 | C>G | 14 | *ADARB1, AGPAT3, AP001048.4, AP001055.6, AP001056.1, AP001057.1,* | dbSUPER, EnhancerAtlas |
|  | lincRNA |  |  | *AP001058.3, C21orf33, ICOSLG, ITGB2-AS1, PWP2, RNU6-859P, SUMO3,* |  |
|  |  |  |  | ***SYNGR1****, TRAPPC10* |  |
| Promoter (2) | 22:39745599 | G>T | 9 | ***SYNGR1*** | dbSUPER, PCHi-C |
|  | (5'Flank) |  |  |  |  |
| Promoter (1) | X:18692747 | G>C | 16 | *PPEF1, RS1* | JEME, PCHi-C |
|  | (5'Flank) |  |  |  |  |
| Promoter (1) | X:30593360 | A>G | 10 | *CXorf21, GK, TAB3-AS1* | dbSUPER, EnhancerAtlas |
|  | (intron) |  |  |  |  |
| Promoter (2) | X:46404008 | G>T | 16 | *CHST7, KRBOX4, RP2, SLC9A7, ZNF674, ZNF674-AS1* | JEME, PCHi-C |
|  | (5'UTR) |  |  |  |  |
| Transcription (11) | 1:55660254 | G>A | 22 | *USP24* | dbSUPER, EnhancerAtlas |
|  | (intron) |  |  |  |  |
| Transcription (11) | 1:204359972 | A>G | 3 | *ATP2B4, DSTYK, ETNK2, GOLT1A, MDM4, PIK3C2B, PLEKHA6, PPP1R15B,* | dbSUPER, EnhancerAtlas, |
|  | (intron) |  |  | *REN, RP11-739N20.2* | JEME |
| Transcription (9) | 2:60703664 | C>T | 18 | *AC009970.1, BCL11A* | dbSUPER, EnhancerAtlas |
|  | (intron) |  |  |  |  |
| Transcription (9) | 2:99106420 | TGGAGTGA>T | 22 | *INPP4A, MGAT4A* | dbSUPER, EnhancerAtlas |
|  | (intron) |  |  |  |  |
| Transcription (11) | 2:160430977 | C>T | 13 | *BAZ2B, CD302, MARCH7, WDSUB1,* ***Y_RNA*** | EnhancerAtlas, JEME |
|  | (intron) |  |  |  |  |
| Transcription (9) | 2:219092185 | C>G | 7 | *AC021016.8,* ***ARPC2*** | dbSUPER, EnhancerAtlas |
|  | (intron) |  |  |  |  |
| Transcription (11) | 2:240152566 | G>A | 7 | *AC017028.1, MIR4269, OTOS* | dbSUPER, |
|  | (intron) |  |  |  | EnhancerAtlas |
| Transcription (11) | 4:8404691 | G>GTTCGGGTGG | 14 | *ACOX3, HTRA3, RNA5SP152, TRMT44* | dbSUPER, EnhancerAtlas |
|  | (intron) | GGACCC |  |  |  |
| Transcription (11) | 4:48235364 | T>G | 10 | *FRYL, NIPAL1, RP11-121C2.2, SLAIN2, TEC, TX* | dbSUPER, EnhancerAtlas, |
|  | (intron) |  |  |  | JEME |
| Transcription (11) | 4:124341327 | G>A | 15 | *AC109357.1, SPRY1* | dbSUPER, EnhancerAtlas |
|  | (intergenic) |  |  |  |  |
| Transcription (10) | 6:42939628 | G>A | 13 | *KLC4, PEX6, PPP2R5D, RPL7L1* | EnhancerAtlas, PCHi-C |
|  | (intron) |  |  |  |  |
| Transcription (9) | 7:73633944 | C>A | 4 | *ABHD11, CLIP2, EIF4H, LAT2, MIR590, RFC2, WBSCR16* | dbSUPER, EnhancerAtlas, |
|  | (intron) |  |  |  | PCHi-C |
| Transcription (9) | 9:117112988 | A>T | 16 | *AKNA, ATP6V1G1, ORM2* | dbSUPER, EnhancerAtlas |
|  | (intron) |  |  |  |  |
| Transcription (11) | 10:80903546 | T>G | 10 | *ZMIZ1* | dbSUPER, EnhancerAtlas |
|  | (intron) |  |  |  |  |
| Transcription (11) | 10:120894443 | G>A | 14 | *FAM45A, MCMBP, PRDX3, RGS10, SFXN4, TIAL1* | dbSUPER, EnhancerAtlas |
|  | (intron) |  |  |  |  |
| Transcription (11) | 10:126359576 | C>T | 4 | *FAM53B, FAM175B, LHPP, METTL10, MIR4296, NKX1-2, RP11-298J20.3,* | dbSUPER, EnhancerAtlas, |
|  | (intron) |  |  | ***Y_RNA****, ZRANB1* | JEME |
| Transcription (10) | 11:93462148 | C>G | 19 | *ANKRD49, C11orf54, MED17, SNORA8, SNORA18, TAF1D, VSTM5* | EnhancerAtlas, JEME |
|  | (intron) |  |  |  |  |
| Transcription (11) | 13:114828114 | T>C | 9 | *CDC16, CHAMP1, GAS6, LAMP1, PCID2, RASA3, UPF3A* | dbSUPER, EnhancerAtlas |
|  | (intron) |  |  |  |  |
| Transcription (10) | 16:2641638 | G>A | 8 | *AC141586.5, CTD-3126B10.1, LOC652276, PDPK1, RP11-20I23.1* | dbSUPER, EnhancerAtlas, |
|  | (intron) |  |  |  | PCHi-C |
| Transcription (11) | 16:72966538 | T>C | 19 | *AC004158.2, HP, RP5-991G20.1, ZFHX3* | dbSUPER, EnhancerAtlas |
|  | (intron) |  |  |  |  |
| Transcription (10) | 18:74830186 | G>A | 4 | *GALR1, MBP* | dbSUPER, EnhancerAtlas, |
|  | (intron) |  |  |  | JEME |
| Transcription (10) | 20:50109645 | G>A | 9 | *MIR1302-5, MIR3194, NFATC2* | dbSUPER, EnhancerAtlas |
|  | (intron) |  |  |  |  |
| Transcription (11) | 22:30665977 | G>A | 14 | *LIF, OSM* | dbSUPER, PCHi-C |
|  | (5'Flank) |  |  |  |  |
| Transcription (10) | X:37662788 | G>A | 7 | *CYBB* | dbSUPER, EnhancerAtlas |
|  | (intron) |  |  |  |  |
| Heterochr/lo (13) | 2:204761623 | G>A | 1 | *CTLA4, NBEAL1, PARD3B, RN7SL670P* | dbSUPER, EnhancerAtlas |
|  | (intergenic) |  |  |  |  |
| Heterochr/lo (13) | 2:219181273 | C>T | 12 | *AAMP, AC021016.6,* ***ARPC2****, ATG9A, CTDSP1, CYP27A1, DIRC3, GPBAR1,* | dbSUPER, EnhancerAtlas |
|  | (intron) |  |  | *PNKD, RP11-378A13.1, SLC11A1, snoU13, STK36, TMBIM1, TNS1, VIL1,* |  |
|  |  |  |  | *ZFAND2B, ZNF142* |  |
| Heterochr/lo (13) | 3:71258273 | G>A | 7 | *FOXP1* | dbSUPER, EnhancerAtlas |
|  | (intron) |  |  |  |  |
| Heterochr/lo (13) | 3:155273205 | T>A | 14 | *PLCH1* | dbSUPER, EnhancerAtlas |
|  | (intron) |  |  |  |  |
| Heterochr/lo (13) | 5:1540197 | C>T | 3 | *CLPTM1L, IRX4, LPCAT1, SLC6A3* | dbSUPER, EnhancerAtlas, |
|  | (3'Flank) |  |  |  | JEME |
| Heterochr/lo (13) | 5:146863866 | C>G | 7 | *DPYSL3, SCGB3A2* | dbSUPER, EnhancerAtlas |
|  | (intron) |  |  |  |  |
| Heterochr/lo (13) | 6:82464722 | T>G | 24 | *FAM46A* | dbSUPER, |
|  | (5'Flank) |  |  |  | EnhancerAtlas, PCHi-C |
| Heterochr/lo (13) | 8:580117 | G>C | 22 | *C8orf42, ERICH1, RP11-43A14.1, TDRP* | dbSUPER, EnhancerAtlas |
|  | (intron) |  |  |  |  |
| Heterochr/lo (13) | 9:80562423 | A>C | 7 | ***CEP78****,* ***GNAQ****,* ***VPS13A*** | dbSUPER, EnhancerAtlas |
|  | (intron) |  |  |  |  |
| Heterochr/lo (13) | 9:80562425 | A>AGGGG | 7 | ***CEP78****,* ***GNAQ****,* ***VPS13A*** | dbSUPER, EnhancerAtlas |
|  | (intron) |  |  |  |  |
| Heterochr/lo (13) | 9:80576284 | G>T | 14 | ***CEP78****,* ***GNAQ****,* ***VPS13A*** | dbSUPER, EnhancerAtlas |
|  | (intron) |  |  |  |  |
| Heterochr/lo (13) | 9:123498896 | T>G | 3 | *MEGF9, PSMD5, STOM* | dbSUPER, EnhancerAtlas |
|  | (intergenic) |  |  |  |  |
| Heterochr/lo (13) | 10:49672255 | A>G | 7 | *ARHGAP22, MAPK8* | dbSUPER, EnhancerAtlas |
|  | (intron) |  |  |  |  |
| Heterochr/lo (13) | 12:8015686 | G>T | 22 | *APOBEC1, C3AR1, CLEC4C, DPPA3,* ***EMG1****, FOXJ2, GDF3, MFAP5, NANOG,* | EnhancerAtlas, JEME |
|  | (intron) |  |  | *NANOGNB, NECAP1, SLC2A3, SLC2A14* |  |
| Heterochr/lo (13) | 12:11801011 | T>G | 18 | *ETV6, PRB2* | dbSUPER, EnhancerAtlas, |
|  | (5'Flank) |  |  |  | PCHi-C |
| Heterochr/lo (13) | 17:62061542 | G>C | 22 | *C17orf72, CTC-264K15.6, CYB561, DCAF7, ICAM2, PSMC5, RP11-214C8.2,* | dbSUPER, EnhancerAtlas |
|  | (intron) |  |  | *SCN4A, SNORA76, SNORD104, TEX2* |  |
| Heterochr/lo (13) | 19:28886062 | G>A | 3 | *AC005307.1, AC005780.1, LOC148145* | dbSUPER, EnhancerAtlas |
|  | lincRNA |  |  |  |  |
| Heterochr/lo (13) | 22:39354242 | T>TGGGGGGGAC | 14 | *APOBEC3A, APOBEC3G, CBX6, SYNGR1* | dbSUPER, EnhancerAtlas |
|  | (intron) |  |  |  |  |
| Repressed (12) | 17:46672043 | C>T | 1 | *HOXB-AS3, HOXB5, HOXB6, MIR10A* | dbSUPER, PCHi-C |
|  | 3'UTR |  |  |  |  |
| Insulator (8) | 4:174338342 | G>C | 14 | *SAP30, SCRG1* | EnhancerAtlas, PCHi-C |
|  | (3'Flank) |  |  |  |  |

^a^Chromatin state annotation of the SNVs/indels according to chromHMM [1, 2]: 1, active promoter; 2, weak promoter; 3, poised promoter; 4 and 5, strong enhancer; 6 and 7, weak enhancer; 8, insulator; 9, transcriptional transition; 10, transcriptional elongation; 11, weak transcription; 12, repressed; 13, heterochromatin, low signal.

^b^ Bold type indicates genes that were targeted in more than one of the pediatric AML cases.

^c^Non coding regulatory variants were annotated by the dbSUPER [3], EnhancerAtlas [4], JEME [5], and the Promoter Capture Hi-C (PCHi-C) datasets [6]. SNV, single nucleotide variant; indel, insertion/deletion; lincRNA, long non-coding RNA.

**REFERENCES**

1. Ernst J, Kellis M. ChromHMM: automating chromatin-state discovery and characterization. Nat Methods. 2012;9:215-6.
2. Ernst J, Kheradpour P, Mikkelsen TS, Shoresh N, Ward LD, Epstein CB, et al. Mapping and analysis of chromatin state dynamics in nine human cell types. Nature. 2011;473:43-9.
3. Khan A, Zhang X. dbSUPER: a database of super-enhancers in mouse and human genome. Nucleic Acids Res. 2016;44:D164-71.
4. Gao T, He B, Liu S, Zhu H, Tan K, Qian J. EnhancerAtlas: a resource for enhancer annotation and analysis in 105 human cell/tissue types. Bioinformatics. 2016;32:3543-51.
5. Cao Q, Anyansi C, Hu X, Xu L, Xiong L, Tang W, et al. Reconstruction of enhancer-target networks in 935 samples of human primary cells, tissues and cell lines. Nat Genet. 2017;49:1428-36.
6. Javierre BM, Burren OS, Wilder SP, Kreuzhuber R, Hill SM, Sewitz S, et al. Lineage-specific genome architecture links enhancers and non-coding disease variants to target gene promoters. Cell. 2016;167:1369-84.e19.

**Supplementary Table 8** Cell lines used in the analysis of variants in regulatory elements

| dbSUPER | | EnhancerAtlas | JEME | PCHi-C |
| --- | --- | --- | --- | --- |
| CD4_Memory_Primary_7pool | | AML_blast | CNhs10837 | Activated total CD4+ T cells |
| CD4_Memory_Primary_8pool | | CD14+ | CNhs10860 | Endothelial precursors |
| CD4_Naive_Primary_7pool | | CD19+ | CNhs10872 | Erythroblasts |
| CD4_Naive_Primary_8pool | | CD20+ | CNhs11045 | Fetal thymus |
| CD4p_CD225int_CD127p_Tmem | | CD34+ | CNhs11049 | Macrophages M0 |
| CD4p_CD25-_CD45RAp_Naive | | CD4+ | CNhs11050 | Macrophages M1 |
| CD4p_CD25-_CD45ROp_Memory | | CD8+ | CNhs11075 | Macrophages M2 |
| CD4p_CD25-_Il17-_PMAstim_Th | | CUTLL1 | CNhs11076 | Megakaryocytes |
| CD4p_CD25-_Il17p_PMAstim_Th17 | | GM10847 | CNhs11350 | Monocytes |
| CD8_Memory_7pool | | GM12878 | CNhs11375 | Naive B cells |
| CD8_Naive_7pool | | GM12891 | CNhs11376 | Naive CD4+ T cells |
| CD8_Naive_8pool | | GM12892 | CNhs11377 | Naive CD8+ T cells |
| CD8_primiary | | GM18505 | CNhs11671 | Neutrophils |
| CD14 | | GM18526 | CNhs11672 | Non-activated total CD4+ T cells |
| CD19_Primary | | GM18951 | CNhs11673 | Total B cells |
| CD20 | | GM19099 | CNhs11675 |  |
| CD34_adult | | GM19193 | CNhs11761 |  |
| CD34_fetal | | GM19238 | CNhs11925 |  |
| CD34_Primary_RO01480 | | GM19239 | CNhs11926 |  |
| CD34_Primary_RO01536 | | GM19240 | CNhs11948 |  |
| CD34_Primary_RO01549 | | HL-60 | CNhs11949 |  |
| CD34_fetal | | Jurkat | CNhs11958 |  |
| DHL6 | | K562 | CNhs11967 |  |
| DND41 | | Kasumi-1 | CNhs11977 |  |
| GM12878 | | ME-1 | CNhs11978 |  |
| HBL1 | | Namalwa | CNhs12002 |  |
| Jurkat | | NB4 | CNhs12010 |  |
| K562 | | NKC | CNhs12022 |  |
| Ly1 | | OCI-Ly7 | CNhs12023 |  |
| Ly3 | | PBMC | CNhs12024 |  |
| Ly4 | |  | CNhs12026 |  |
| MM1S | |  | CNhs12105 |  |
| RPMI-8402 |  | | CNhs12495 |  |
| Toledo |  | | CNhs12496 |  |
|  |  | | CNhs12497 |  |
|  |  | | E029 |  |
|  |  | | E030 |  |
|  |  | | E031 |  |
|  |  | | E032 |  |
|  |  | | E033 |  |
|  |  | | E034 |  |
|  |  | | E037 |  |
|  |  | | E038 |  |
|  |  | | E039 |  |
|  |  | | E040 |  |
|  |  | | E041 |  |
|  |  | | E042 |  |
|  |  | | E043 |  |
|  |  | | E044 |  |
|  |  | | E045 |  |
|  |  | | E046 |  |
|  |  | | E047 |  |
|  |  | | E048 |  |
|  |  | | E062 |  |

More information about additional cell lines used in the databases are provided at https://asntech.org/dbsuper/download.php (dbSUPER), http://enhanceratlas.org/download.php (EnhancerAtlas), http://yiplab.cse.cuhk.edu.hk/jeme/ (JEME). In the PCHi-C dataset, all cells were primary human hematopoietic cells [1].

**REFERENCES**

1. Javierre BM, Burren OS, Wilder SP, Kreuzhuber R, Hill SM, Sewitz S, et al. Lineage-specific genome architecture links enhancers and non-coding disease variants to target gene promoters. Cell. 2016;167:1369-84.e19.

**Supplementary Table 9** Primers used for RT-PCR sequencing verification of WGS-identified fusion genes

| Fusion gene | Fusion | Primer 1 | Fusion | Primer 2 |
| --- | --- | --- | --- | --- |
|  | partner 1 |  | partner 2 |  |
| *PLEKHA5-ADAMTS20* | *PLEKHA5* | TTCGTCACGTATCCCGTTTT | *ADAMTS20* | ATGAGGGAACCGAGGCTTA |
| *RAB11FIP2-NEURL4* | *RAB11FIP2* | GGATGTGGGTGTCTTTCCTC | *NEURL4* | GTACTGCCTCCATCCTGAGC |
| *TCF3-HOXB9* | *TCF3* | GCTGGCCTCAGGTTTCAC | *HOXB9* | TCCAGCGTCTGGTATTTGGT |

RT-PCR, reverse transcription polymerase chain reaction; WGS, whole genome sequencing.

**Supplementary Table 10** Primers used for Sanger sequencing verification of single nucleotide variants

| Gene | Genomic | Nt change | Forward sequence (5' to 3') | Reverse sequence (5' to 3') |
| --- | --- | --- | --- | --- |
|  | position |  |  |  |
| *ADAMTS17* | 15:100514744 | C>T | CAAGTCCACGCTCATGTTCT | TGCATCCCTGTGACAGAAAG |
| *AGAP1* | 2:236949450 | G>A | GGGTGCTTACGCCTTGTATT | TCTGCCAAACAGGACAGATG |
| *ALDH1B1* | 9:38395959 | G>A | CAATGAATGGCAAGATGCAG | TCTCGAGTGAGGCCAAGTAGA |
| *APLP1* | 19:36364595 | G>A | GTGGATCAGGGTGGATTCTG | GGCTGAGATAGGTGGGAAGG |
| *ARHGAP21* | 10:24874254 | G>A | ACTTCCCTCGGTGCAACTTA | TGACGAGAGAAGCGAACTCA |
| *ARID3A* | 19:960104 | G>C | ACATGGTTCCCACACCTGAG | GTTTCTGTGACCTCCCTCCA |
| *ASB5* | 4:177142730 | G>A | GTTGGGGATGGAAGACATGA | TGTGCACATTTTCCTACGTATTTC |
| *ASCC3* | 6:101090558 | A>G | TCAAATCAGCATTCACCATGT | GTATCCTCAGAAGGGCACCA |
| *ATP5B* | 12:57036567 | C>T | CCTCTCAAGCCTTCCCTCTT | CTGTGGTCGTTTCTCCGATT |
| *ATP10D* | 4:47560126 | C>A | GCCTGCAACCTGTGTTATGA | TGTCGGACCACAACAGACAT |
| *ATRNL1* | 10:117059569 | G>A | AAAACTCATTAGAAAGGCAGCTT | ACTGCAGCCCTTTCCAGATA |
| *BCORL1* | X:129150029- | incC | AAGGTGGATGGTGATGTGGT | CTGCTGCTCTTCACTGTCCTT |
|  | 129150030 |  |  |  |
| *BCORL1* | X:129150031- | insC | AAGGTGGATGGTGATGTGGT | CTGCTGCTCTTCACTGTCCTT |
|  | 129150032 |  |  |  |
| *C6orf120* | 6:170103039 | G>T | GAGCGAGTTCGAGATGAAGG | GCTTCACAGATGGAGGGTTT |
| *CACNA1E* | 1:181453118 | A>T | GCCTACAAGCAGACGAAAGC | GGGGGATACATTCCATGCTA |
| *CAMK2G* | 10:75602244 | G>C | AGGGGAGCTTCCTTCTGAAA | GTATCGGTGCCAGGACTGTG |
| *CCND1* | 11:69466027- | delCGGGACGTG | GCTGCAGGCCCCTTCTAA | CGCTCAGGGTTATGCAAATA |
|  | 69466035 |  |  |  |
| *CHD3* | 17:7806600 | G>C | CAGCAGCCTTCTTTCCTGAG | AACCAGGTGTGTCAGCATCA |
| *CHD4* | 12:6696937 | T>G | CCACATCCCCAAAGTCAGTT | TTAGCAGAGCTCACCGGATT |
| *CLDN17* | 21:31538860 | C>A | CAATTCATCCAGAGCCCTTC | CCACTCCGAATTGAACCAGT |
| *CLTC* | 17:57763155 | G>A | AGGCCAGATGTCGTCCTAGA | CAAAAGAACAGGAATATTTTATGATCG |
| *CREBBP* | 16:3817733- | delGCAGC | ATGGCAGGCAAGAAAGGTAA | GAACCAATGGAAGTGGATGAA |
|  | 3817737 |  |  |  |
| *DHX15* | 4:24572314 | C>G | TTCACCTAAAATGCAGAACCAA | TACCAGGACCCAAGAGAGGA |
| *ECT2L* | 6:139164189 | G>A | TCTTCCTCTTGTTTCTGAAGGA | TCAGCGTCCCATAAGTAGCA |
| *EPB41L4B* | 9:111956657 | C>A | CGAGCTGACATTCTGGACCT | AATCCCCATCCCTGGTCTT |
| *FAM179B* | 14:45475242 | G>C | GCACTTGTGACTATAACTCCCTCTT | CATCAGGCTCTTTCTTTTTGTG |
| *FAM184B* | 4:17710654 | C>T | TCACTTATCTTGCGGCCTCT | CAGCAGCTGAGCAAGGACTA |
| *GAD2* | 10:26581912 | C>T | TGTAGCCTCAGCACACAAATG | AGGAGTCCACAGACGGGAAT |
| *GALNT18* | 11:11470391 | C>T | CTGACTCTGCTCACCCACTG | TGCACTCTGACCCACTGTCT |
| *GJB1* | X:70443777 | G>T | GGCTCTCGGTCATCTTCATC | TCTTCACCTCCTCCAGGTGT |
| *GRIK2* | 6:102337644 | G>A | GCAGTTGCTCCACTGGCTAT | GCCCACAAAGACAGGACAGT |
| *H3F3C* | 12:31944864 | C>A | CACTTTTCGCATTCATCCTG | ACGAAAGCTGCCAGGAAAAG |
| *H3F3C* | 12:31944836 | G>A | CACTTTTCGCATTCATCCTG | ACGAAAGCTGCCAGGAAAAG |
| *H3F3C* | 12:31944789 | G>C | CACTTTTCGCATTCATCCTG | ACGAAAGCTGCCAGGAAAAG |
| *HPCA* | 1:33354852 | G>T | GAGTTCATCATTGCGCTGAG | ATATCCCCTGCCACCACAT |
| *JAK3* | 19:17948006 | C>T | CAGCCTAGACTTGGGTGAGG | ACCTGTGGCCAGGTGTTC |
| *KANSL1* | 17:44117112- | insA | TCACTGATACTCTGGGGGACTT | CATTTCCAGAGCATGCTGAA |
|  | 44117112 |  |  |  |
| *KIT* | 4:55589771- | delGAC | TGTAGGGATTAGAGAGGGAGTGA | AGTCCTTCCCCTCTGCATTA |
|  | 55589771 |  |  |  |
| *KLHL30* | 2:239049951 | G>A | AGTTCGGGGAGCAGCAAG | GCTCTGAGGCCAGCAGTT |
| *KMT2C* | 7:151845262 | C>T | CCCATCCTTCTCCTCAATGG | ATGTTCAGCGTGATGAGGTG |
| *L3MBTL1* | 20:42157329 | G>A | GGGGTGAAGGTAGAGCTTGG | CCTGAGGTTGGTGGTGTGT |
| *LARP1* | 5:154181828 | G>T | GCCTGGTCTGATGAGGAATC | TCCAAGCCTCACCTTGATCT |
| *LMO2* | 11:33886176 | C>T | ACACACCGACTGCAGATGTC | AGAACATTGGGGACCGCTAC |
| *LRRC46* | 17:45912764 | C>T | AGACATGGTCTTGGGGCTTT | TTGCTGGTCCTTGACTGACA |
| *MACF1* | 1:39918368 | C>T | ACCCTTTCCCTAGCATGTCC | AAACCCCAAGGCTGGATTAC |
| *MN1* | 22:28146926- | delTCCAACAG | GTAAGGTTGAGGGGGAAGGA | GACCTCCCTGCAAACAAGG |
|  | 28146941 |  |  |  |
| *MN1* | 22:28146918- | insA | GTAAGGTTGAGGGGGAAGGA | GACCTCCCTGCAAACAAGG |
|  | 28146919 |  |  |  |
| *MN1* | 22:28146922- | delATT | GTAAGGTTGAGGGGGAAGGA | GACCTCCCTGCAAACAAGG |
|  | 28146924 |  |  |  |
| *MN1* | 22:28195720 | C>T | CCCCACTGAACCTCTCAAAG | AGGGTGACGAACCAAGGAG |
| *MUC7* | 4:71346978 | T>C | CCATCAGCTTCCACCAAAAT | CGGATGATGGGTCTAGGGTA |
| *MUC17* | 7:100679919 | A>C | CCAGCTCTGCAATCAGCAC | GGAGACGAAGTGGCTTCA |
| *MYLK* | 3:123337540 | C>T | CATCCAGCCACACCCTATTC | GCGAGAACAACGAGTGAAGA |
| *MYLK3* | 16:46771797 | C>T | AGAGCTCCCCTTACCTTGGA | CCAGGGAGATGGTGTTCCT |
| *NCKAP5* | 2:133542104 | C>A | TTCGGGCTCCATTAGTTTTG | GGACCCAAGGCAGAACATAC |
| *NCKAP5L* | 12:50195748 | C>A | GAGTGAAAGGAGCGCAGATG | CTCCCACCAGGGGTGATAA |
| *NCOR1* | 17:16049709- | insG | TCCGAGAAACACCCAAGAAT | ATGGAGGCATGGGAGAAAA |
|  | 16049710 |  |  |  |
| *NCOR1* | 17:16012202 | C>T | CCTTCGCTGTCTTCTGGATT | TGACCTCCTTGTCTGGTGTG |
| *NDN* | 15:23931856 | C>T | CACGTAGATGAGGCTCAGGA | TGGTTTCCAGACATGGTGAA |
| *NKAP* | X:119077423 | G>A | GACTGACGAGGAGGCAGAAG | GGAACCAAGGCAACAGACAT |
| *NRAS* | 1:115256530 | C>A | TGGTAACCTCATTTCCCCATA | GCAATTTGAGGGACAAACCA |
| *NRAS* | 1:115256529 | A>G | TGGTAACCTCATTTCCCCATA | GCAATTTGAGGGACAAACCA |
| *NRG2* | 5:139231294 | G>A | CTGGCTAAGCGGGGAATAG | ATCTGGCCTTCCCAGACAC |
| *OR1L3* | 9:125438260 | G>T | TGCAGCCTTTGTCCAGCTAT | CGAGGTGAAGCTACAAATGGT |
| *OR1Q1* | 9:125377798 | G>A | CTGCATCATCCTGGTGGTC | CATGAAGCCTCCCTTGACAT |
| *OR4A15* | 11:55135405 | G>T | AACGTGGTCGATAAGGCATC | CCCTCAGGGTTCTGTGTGAG |
| *OR8H1* | 11:56058050 | C>A | TGTCGTATGTGTCCATGCAG | AATGGCCTATGATCGCTACG |
| *PDLIM5* | 4:95497118 | G>A | GCTAATGCCAATCTTAGTGCTG | GGAGAGGAATGTAGAGTATTTCCTG |
| *PDZD4* | X:153074043 | G>A | GAGTGCCACTGTCCACCAG | GAGTGCCACTGTCCACCAG |
| *PFAS* | 17:8172052 | G>A | GTCAACTGCTGGCTCTGCTC | CTTCCTTGGGCCTGACCT |
| *PHLDB1* | 11:118502116 | C>T | GGAGCTTCCCAGCATTGG | CAGGTCCAACTTCAGCCTCT |
| *PIGC* | 1:172410982 | A>G | GGAACCTGGACAAGTCTTCCT | GCCCATGTTGCAGAAGAAAC |
| *PLCE1* | 10:96006214 | C>A | CCACCCTGGCATTGATATAC | GTACTGTTTCCGCAGCCACT |
| *PRELID1* | 5:176731732 | G>C | CTGACACCTGCAGCAAACAT | GGTGCAGAATCACAACAGGA |
| *PTPN5* | 11:18754162 | C>T | ACTTGGGGGACCTGTTCTCT | GGATTGTATGGCAGCCTGAT |
| *RAC2* | 22:37637633 | C>G | GTGCCCACACAGGGTCTG | CAGAAACCTCCCCACGTCT |
| *RASL11A* | 13:27847335 | G>A | GTCGATTCCCTGTCCAAATG | GGGATGATGGAGGCTCTTCT |
| *RBM12B-AS1* | 8:94752638 | T>G | TAGATCTGGGGGCAGTTCAC | TCGATTCTACAGCCCAAAGC |
| *RNF148* | 7:122341919 | C>T | GAGAAGCTGAAATTTTCTCTCTACA | TCAAAGAAGGGGATGAGGAA |
| *RPH3AL* | 17:65506 | C>T | CACCGACCAGTCTCCAGTTT | GACTGAGATCACCCCGTCTC |
| *RPL10A* | 6:35438435 | A>G | CTCTTCCCTCCTCCCAGGT | ACCCTCAATCACAGGCAGAC |
| *SAGE1* | X:134994097 | C>T | GCAGGTGACCCACCAGTTAC | ATGAGCTCAAGAGGCAGCTC |
| *SLIT2* | 4:20620505 | G>C | TTGTCGAGGGGAAAGGATAA | CTGCCGGGAGTGTGTTTAG |
| *SPATA31A1* | 9:39361126 | G>A | CCAGAAGGAGCAAACTGGTG | GGAGGCTGTTTCTTTGATGG |
| *SYCP2* | 20:58468227 | C>A | TCCTTTTCCTCCAAATCTGTT | CAATCCGTTCATCCCAATATG |
| *TEDC2* | 16:2510988 | C>T | AGGCACTGGAGAAGGCTGTA | ATCCCCCACTGACAGCAG |
| *TM9SF4* | 20:30738461 | G>A | GAGTCCTCCTGTCAGCCTGT | AAACACCCTGTGGGAAAATG |
| *TMED6* | 16:69383461 | C>T | CCCCCAAATCGTCAGTAGAC | CGTGGGACGTCAATCTTACC |
| *TMEM132E* | 17:32964671 | G>T | ACGAGGAGGACCCCACTTAT | CAGCACAAAAACGATGCAGT |
| *TTC28* | 22:28490212 | A>G | CTCCCGGACACTGGCAAT | GGCATGCTTGTCAAAGAAGTC |
| *UBE2D2* | 5:138979975 | C>T | TTTCACCAGCAGGACAGAGA | TCATGGGCCACTAAGGGTTA |
| *UNC5D* | 8:35608226 | G>A | CCTTTCCCAACAGGAAGTGA | AACCCACTGGGCAGTTACAG |
| *ZC3H12A* | 1:37947391 | G>A | GGTGTTCACACCATCACGAC | TCCCGGTCTGATTTCTCCTA |
| *ZFAT* | 8:135669869 | delA | TCAACAGGTGTGTCAGAAGGT | TGTCTTTGGCAATTGGAAGT |
| *ZNF251* | 8:145947604 | C>T | CGCTGTGAACTTTCTGATGC | CAAAGCCTTTCGTCGGAGT |
| *ZNF365* | 10:64136628 | G>A | CCAGAAAACTGCGGAACTGT | TTTGCTTGTAAGGCGATTGG |
| *ZNF544* | 19:58758142 | G>A | GGACATGGTTCTTGGTGTCC | GAGGCTTCCTCCAAACAAGG |

Nt, nucleotide.

**Supplementary Figure 1**


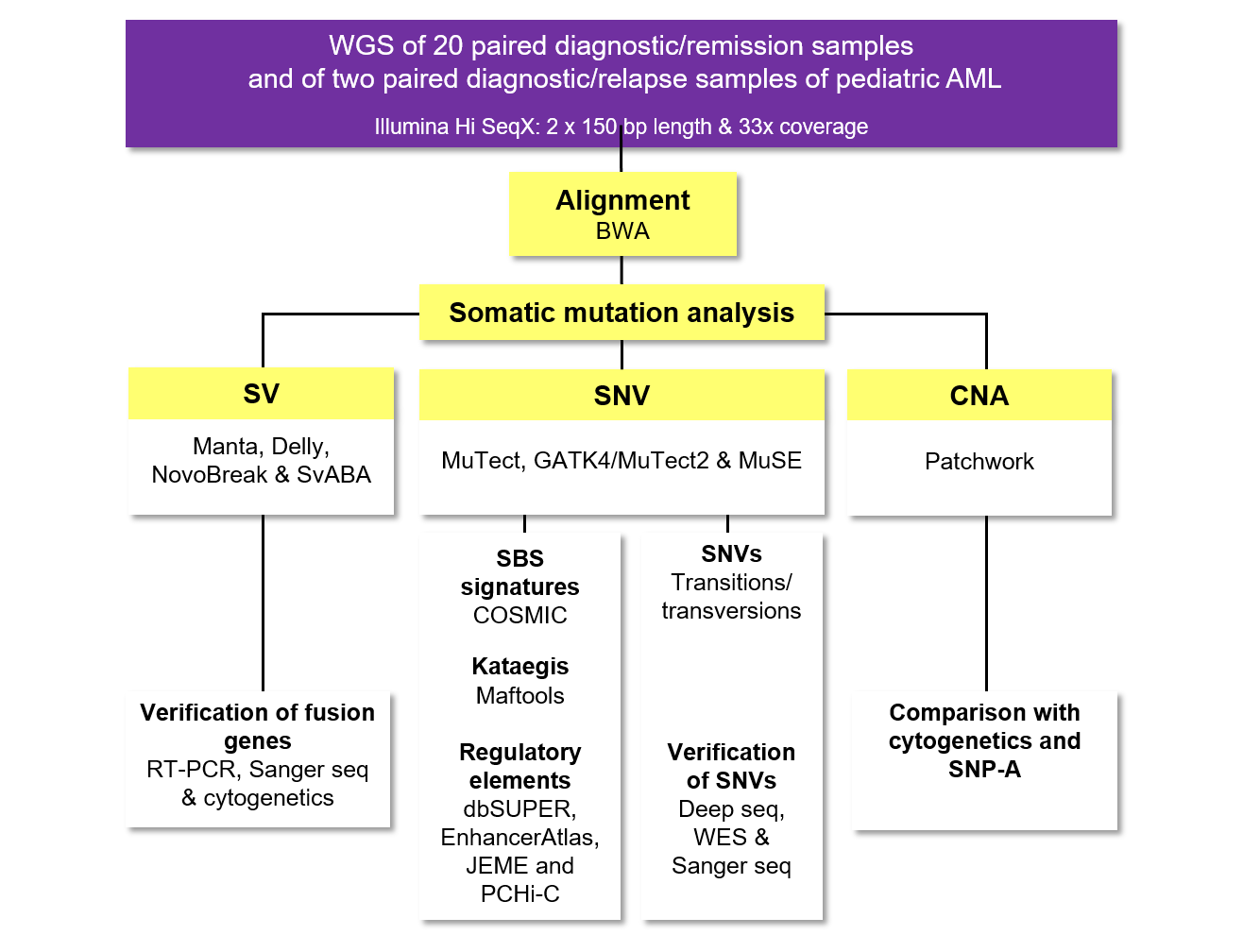
Overview of the whole genome sequencing (WGS) and bioinformatic analyses of the pediatric acute myeloid leukemia (AML) cases. After alignment to the reference genome GRCh37 (hg19), the analytical tools listed in the figure were applied to identify copy number abnormalities (CNAs), single nucleotide variants (SNVs), and structural variants (SVs), including fusion genes. The SNV data were used to ascertain transition and transversion types, kataegis, single base substitution (SBS) mutational signatures, and variants in non-coding regulatory elements.

BWA, Burrows-Wheeler Aligner; RT-PCR, reverse transcription polymerase chain reaction; seq, sequencing; SNP-A, single nucleotide polymorphism array; WES, whole exome sequencing.

**Supplementary Figure 2**


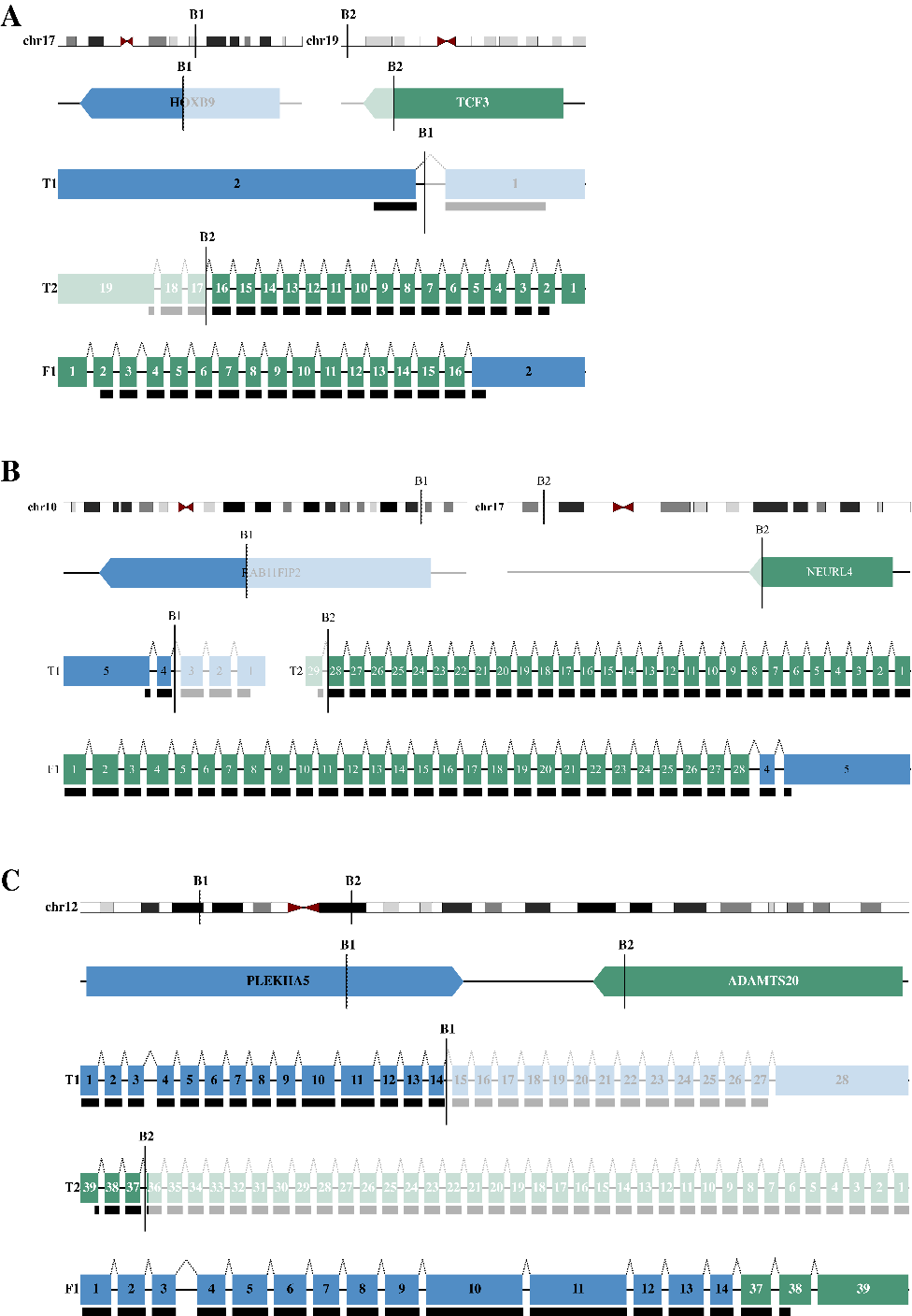


Novel fusion genes in AML. **A** t(17;19)(q21;p13) with *TCF3*-*HOXB9* (case 3). **B** t(10;17)(q26;p13) with *RAB11FIP2-NEURL4* (case 24). **C** inv(12)(p12q12) with *PLEKHA5-ADAMTS20* (case 14). B1 and B2 indicate the breakpoints; T1, transcript 1; T2, transcript 2; F1, fusion transcript of T1 and T2; the numbers indicate exon number of the fused genes.

**Supplementary Figure 3**


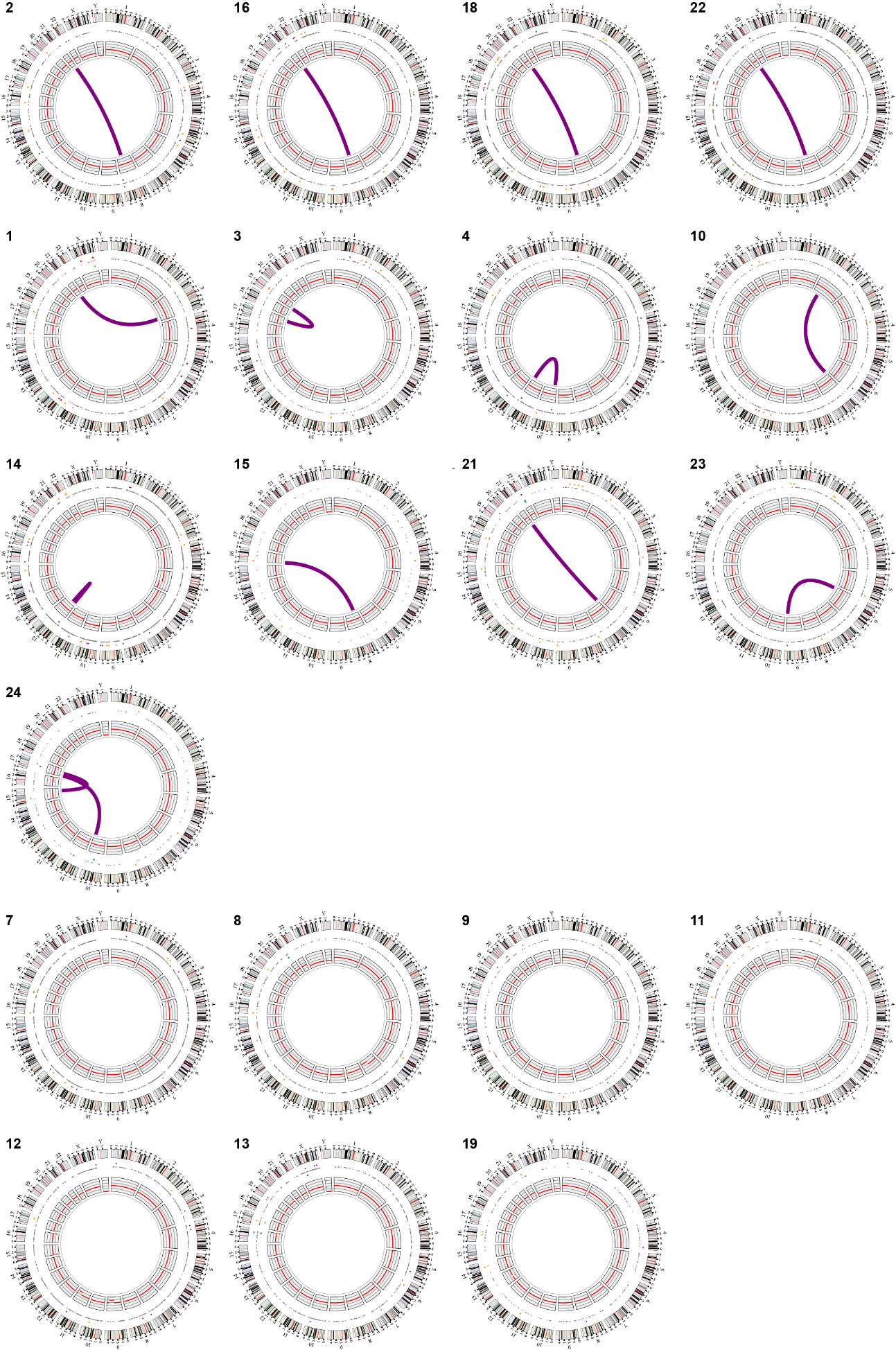


Circos plots of somatic mutations in 20 pediatric acute myeloid leukemias. The top four rows show cases with fusion genes (row 1: *RUNX1-RUNXT1*-positive cases). Cases without fusion genes are shown on the two bottom rows. The outermost circles indicate chromosomes 1-22, X, and Y. Missense mutations are indicated by purple dots, insertions/deletions by green dots, single nucleotide variants in regulatory elements by orange dots, synonymous mutations by grey dots, alternative splice variants by black dots, copy number abnormalities by red lines, and fusion genes by purple lines.

**Supplementary Figure 4**


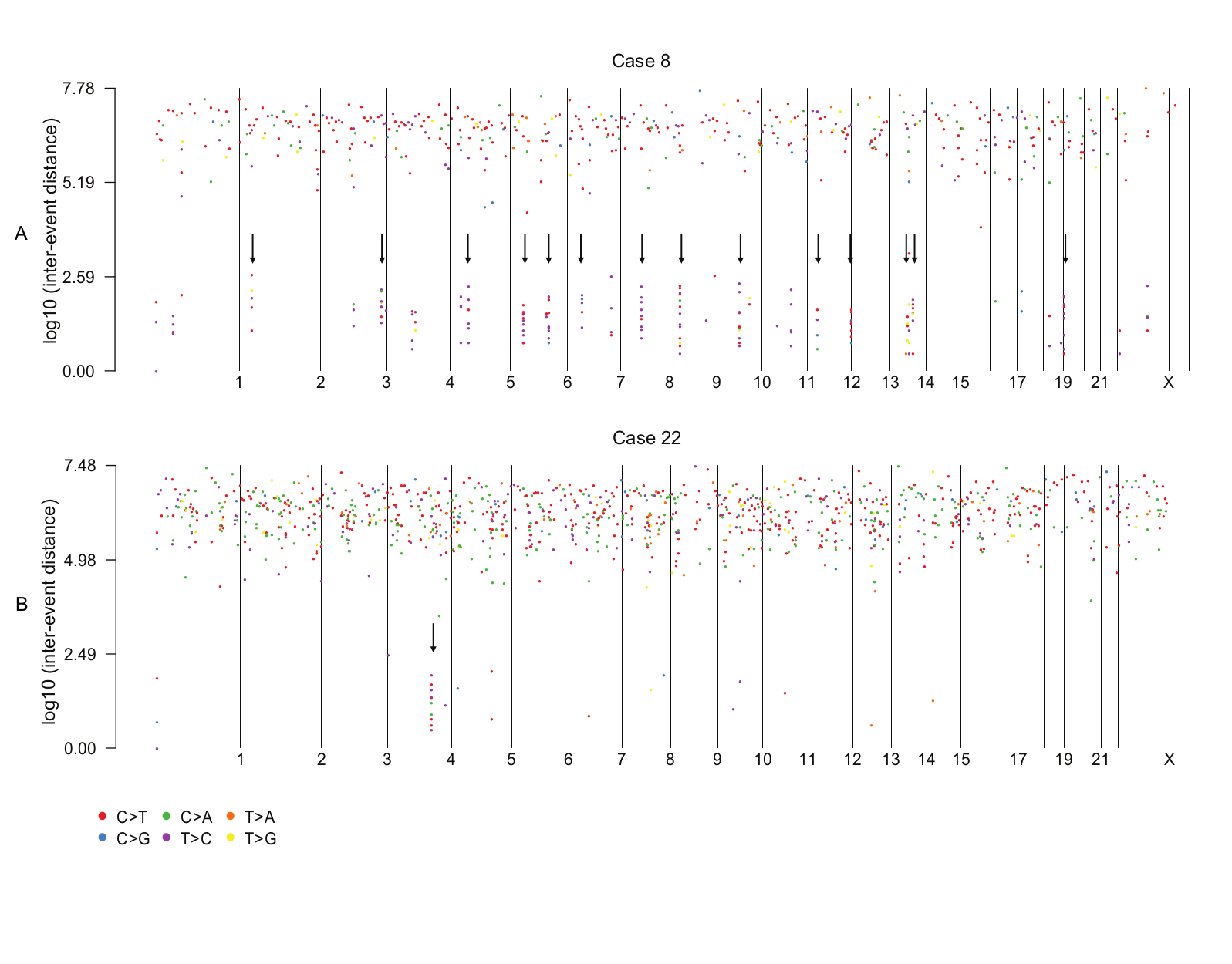


Whole genome overview of the single nucleotide variants (SNVs) in the two pediatric acute myeloid leukemias with kataegic regions (arrows), with the types of transition/transversion indicated by different colors. **A** Case 8 harbored 14 kataegic regions on 12 different chromosomes with 5-13 SNVs within regions sized between 0.08 and 1.9 kb (median 0.37 kb) and with a median average distance between the SNVs of 59 bp (range 17-464 bp). Seven of the kataegic regions were intergenic, five occurred within introns of the *ASPH*, *GOLGA*, *MAP3K13*, *NUDCD3*, and *SMOC1* genes, and two within exons of the *DSE* and *H3F3C* genes. **B** Case 22 displayed one 0.24 kb non-coding region with kataegis harboring 11 SNVs (median distance between the SNVs of 22 bp).

**Supplementary Figure 5**


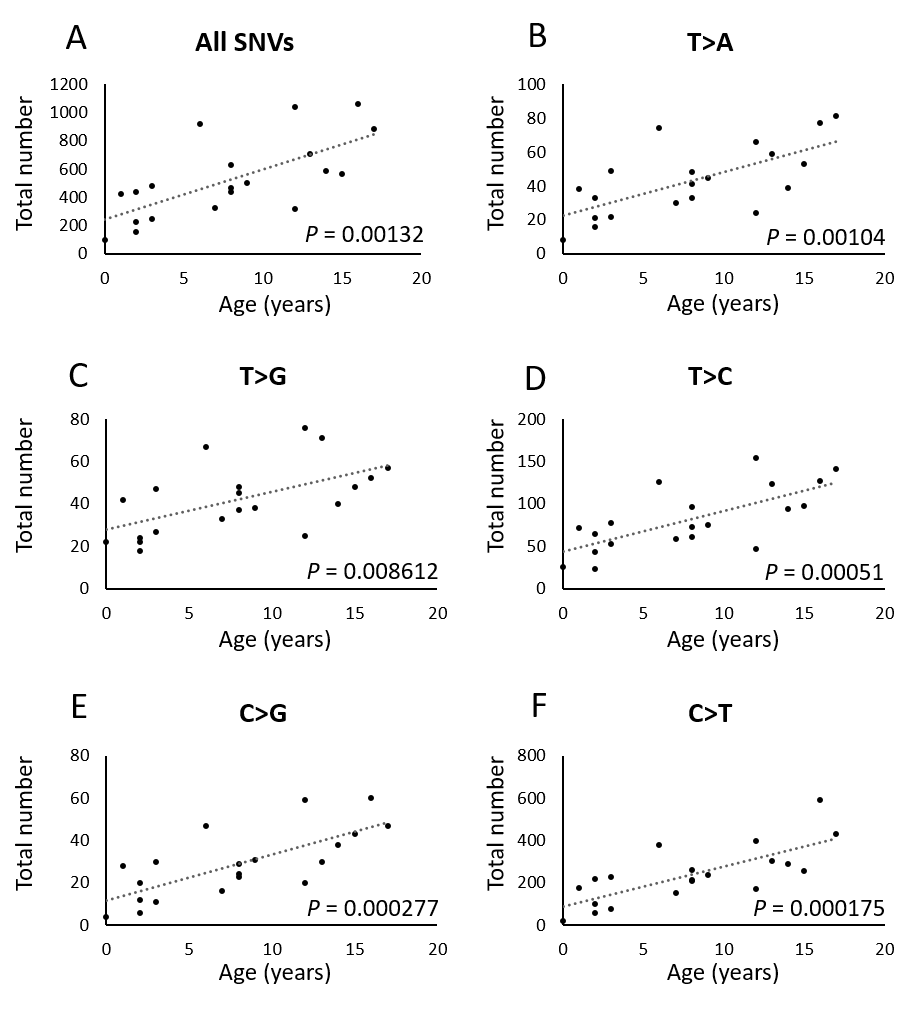


**A** Total number of single nucleotide variants (SNVs) in relation to age. **B-F** Correlations between different transition/transversion types (except C>A; not shown) and age. The *P*-values are based on linear regression analyses.

**Supplementary Figure 6**


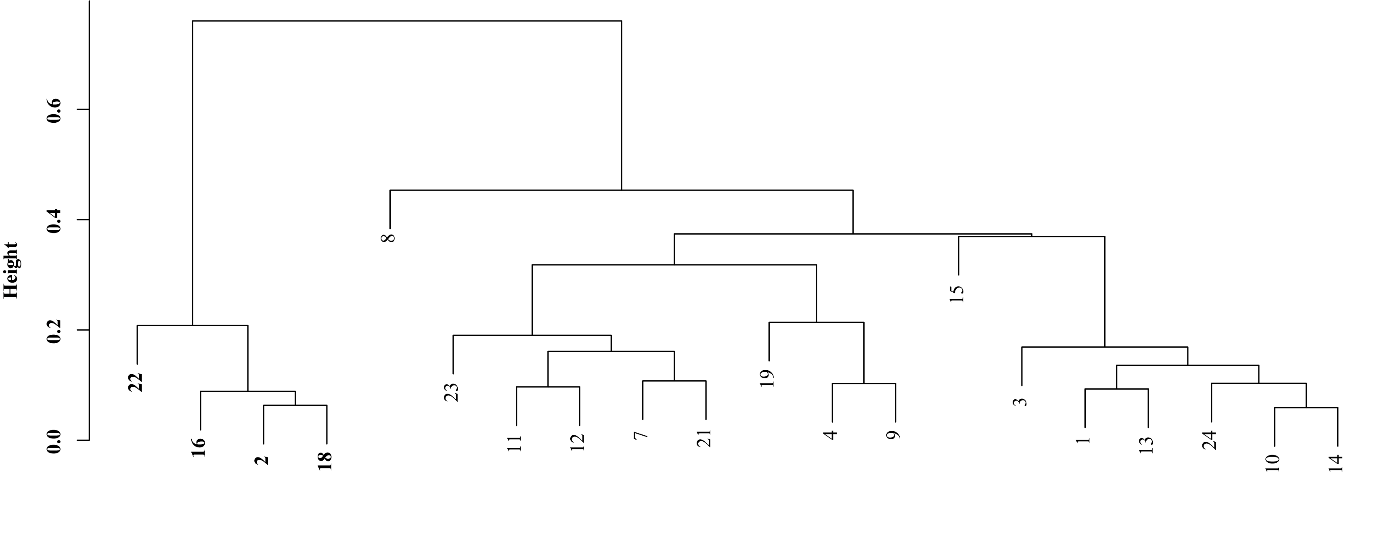


Hierarchical clustering of the relative contributions of the five most common single base substitution mutational signatures (SBS1, SBS18, SBS32, SBS37, and SBS39) in the 20 pediatric acute myeloid leukemias. The *RUNX1-RUNXT1*-positive cases, displayed in bold type (#2, 16, 18, and 22; Supplementary Table 1), clustered together in a separate branch, possibly due to the high frequency of C>A transversions in this AML subtype (two-tailed Mann-Whitney U test *P* = 0.0008256).

**Supplementary Figure 7**

**
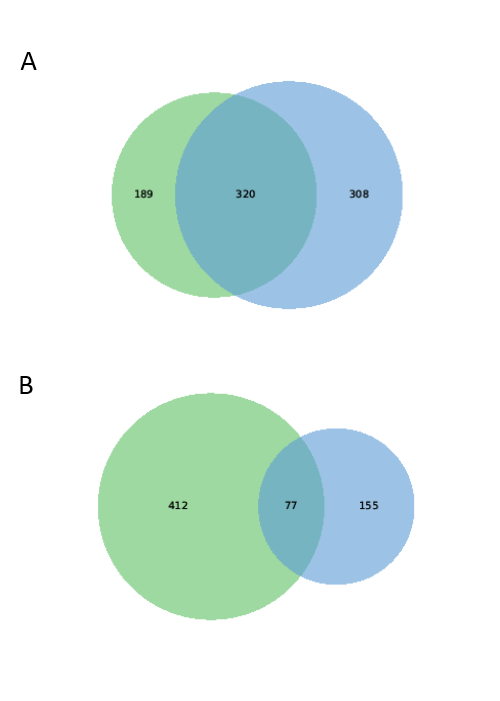
**

Venn diagrams of number of single nucleotide variants (SNVs) and insertions/deletions (indels) present at diagnosis (green) and at relapse (blue). **A** In case 1, 509 SNVs/indels were identified at diagnosis and 628 at relapse; of these, 320 were identical. B In case 2, 489 (diagnosis) and 232 SNVs/indels (relapse) were detected; of these, 77 overlapped.

**Supplementary Figure 8**

**
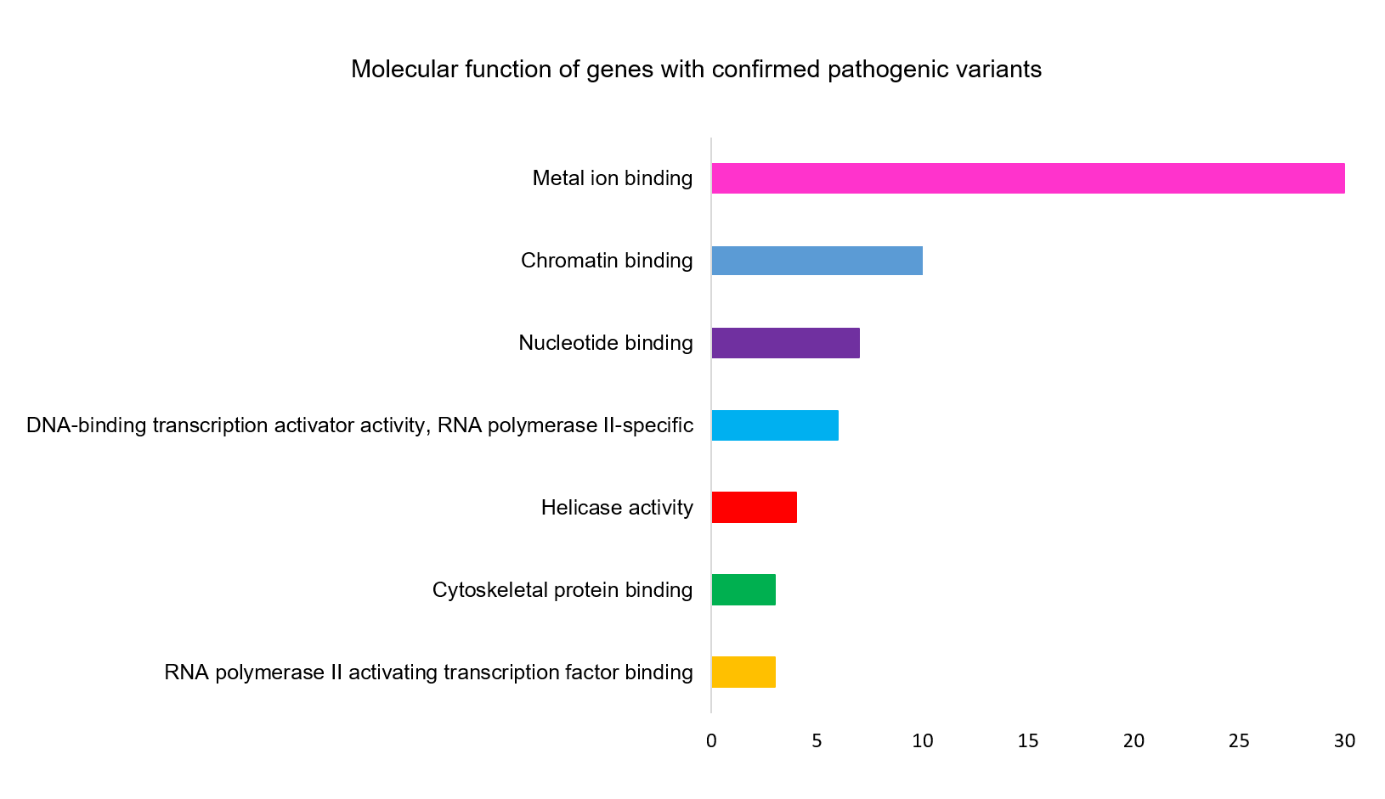
**

Gene ontology (GO) data on molecular functions were available for 43 of the 84 genes with variants considered pathogenic, either by resulting in truncation or by being classified as such by SIFT and/or PolyPhen. Fourteen of the 43 genes had more than one GO-annotated function, making the total number of functions 63.
